# Specific roles for dendritic cell subsets during initiation and progression of psoriasis

**DOI:** 10.15252/emmm.201404114

**Published:** 2014-09-12

**Authors:** Elisabeth Glitzner, Ana Korosec, Patrick M Brunner, Barbara Drobits, Nicole Amberg, Helia B Schonthaler, Tamara Kopp, Erwin F Wagner, Georg Stingl, Martin Holcmann, Maria Sibilia

**Affiliations:** 1 Department of Medicine I, Comprehensive Cancer Center, Institute of Cancer Research, Medical University of ViennaVienna, Austria; 2 Department of Dermatology, Division of Immunology, Allergy and Infectious Diseases, Medical University of ViennaVienna, Austria; 3 BBVA Foundation–CNIO Cancer Cell Biology Programme, Spanish National Cancer Research Centre (CNIO)Madrid, Spain

**Keywords:** AP-1, IL-23, Langerhans cells, plasmacytoid dendritic cells, psoriasis

## Abstract

Several subtypes of APCs are found in psoriasis patients, but their involvement in disease pathogenesis is poorly understood. Here, we investigated the contribution of Langerhans cells (LCs) and plasmacytoid DCs (pDCs) in psoriasis. In human psoriatic lesions and in a psoriasis mouse model (DKO* mice), LCs are severely reduced, whereas pDCs are increased. Depletion of pDCs in DKO* mice prior to psoriasis induction resulted in a milder phenotype, whereas depletion during active disease had no effect. In contrast, while depletion of Langerin-expressing APCs before disease onset had no effect, depletion from diseased mice aggravated psoriasis symptoms. Disease aggravation was due to the absence of LCs, but not other Langerin-expressing APCs. LCs derived from DKO* mice produced increased IL-10 levels, suggesting an immunosuppressive function. Moreover, IL-23 production was high in psoriatic mice and further increased in the absence of LCs. Conversely, pDC depletion resulted in reduced IL-23 production, and therapeutic inhibition of IL-23R signaling ameliorated disease symptoms. Therefore, LCs have an anti-inflammatory role during active psoriatic disease, while pDCs exert an instigatory function during disease initiation.

## Introduction

Psoriasis is a frequent pathology of the skin affecting about 2% of the total Western population. It is characterized by inflamed lesions that display abnormal keratinocyte proliferation and differentiation as well as prominent immune cell infiltration. Both the innate and the adaptive immune system play a role in the pathomechanism of psoriasis (Nestle *et al*, 2009), and several cues point to a role of keratinocytes in psoriasis etiology (Nickoloff, 2006). In human psoriatic skin, an overall increase of dendritic cells (DCs) has been found both in the epidermis and in the dermis (Lowes *et al*, 2005; Wagner *et al*, 2010). DC types that are normally absent in healthy skin, such as TNF and iNOS-producing DCs (Tip-DCs) (Lowes *et al*, 2005), slanDCs (Schakel *et al*, 2006), and plasmacytoid DCs (pDCs) (Nestle *et al*, 2005), have been shown to infiltrate predominantly the dermal compartment of psoriatic skin. Whereas little is known about the roles of the different DC subsets in psoriasis, recent reports indicate that DCs are an important source of IL-23, a cytokine that seems to have, along with TNF-α and IL-17, a central role in psoriasis pathology (Brunner *et al*, 2013; Di Cesare *et al*, 2009; Gunther *et al*, 2013; Wohn *et al*, 2013). Likewise, polymorphisms in the IL-23 receptor (IL-23R) have been associated with psoriasis (Di Meglio *et al*, 2013), and blocking IL-23 is successful in the treatment of psoriasis (Crow, 2012). Recent findings indicate that inhibitors of TNF-α signaling, which are similarly useful in therapy, seem to function via blockage of DC-derived IL-23 (Brunner *et al*, 2013; Gunther *et al*, 2013). IL-23 promotes the maintenance of T cells producing IL-17 and IL-22, which are abundant in and contribute to many of the hallmarks seen in psoriasis. In psoriatic skin, these are constituted by both CD4^+^ and CD8^+^ TCRαβ^+^ T cells, as well as γδ T cells, and the recently discovered innate lymphoid cells (ILCs) (Dyring-Andersen *et al*, 2014; Lowes *et al*, 2014).

pDCs have been detected in low numbers even within uninvolved skin of psoriatic patients and have therefore been implicated in the conversion of healthy into lesional skin (Nestle *et al*, 2005). In mice engrafted with human psoriatic skin, the formation of lesions could be inhibited by pre-treatment of mice with antibodies that blocked pDC-specific type I IFN secretion (Nestle *et al*, 2005). Therefore, targeting pDCs as a therapeutic measure against clinically manifest psoriasis has been discussed. Another DC subset that has been suspected to be involved in psoriasis are Langerhans cells (LCs), which are constitutively resident within the epidermis. In contrast to most other immune cells that recycle from the bone marrow, the LC compartment renews under steady-state conditions from an epidermis-resident precursor population that is maintained from an early embryonic age throughout life (Chorro *et al*, 2009; Hoeffel *et al*, 2012; Merad *et al*, 2008). In addition, severe inflammation may provoke additional recruitment of a developmentally unrelated LC precursor from the bone marrow (Merad *et al*, 2008; Nagao *et al*, 2012). While LCs are the only DCs present within healthy epidermis, at least four different types of DCs are present in murine dermis (Tamoutounour *et al*, 2013), among them a subset of DCs that expresses Langerin, termed Langerin-positive dermal DCs (Lan^+^ DDCs). In humans, a counterpart for Lan^+^ DDCs exists, but lacks Langerin expression, and is identified by expression of CD141 (Haniffa *et al*, 2012). In mice, Lan^+^ DDCs can be discriminated from LCs by their additional expression of the αE integrin (CD103) (Merad *et al*, 2008). The role of LCs and Lan^+^ DDCs could be studied using diphtheria-toxin (DT)-based mouse models that express either the DT receptor (DTR) or DT under the control of the Langerin promoter, thus allowing inducible or constitutive depletion of LCs and Lan^+^ DDCs, which are herein mentioned as Lan^+^ (Lan^+^) APCs. These studies demonstrated that dependent on the context, LCs could act either pro- or anti-inflammatory (Bobr *et al*, 2010; Igyarto *et al*, 2011; Ouchi *et al*, 2011; Romani *et al*, 2012; Shklovskaya *et al*, 2011), while Lan^+^ DDCs have proinflammatory roles in most settings (Bedoui *et al*, 2009; Romani *et al*, 2012; Seneschal *et al*, 2014).

Psoriasis etiology is linked with an array of predisposing genes located within several psoriasis susceptibility regions (PSORS). Jun and JunB are members of the activator protein-1 (AP-1) family and act in a heterodimeric fashion together with other AP-1 members. They are located within the susceptibility regions PSORS7 (*Jun*) and PSORS2 (*Junb*) (Schonthaler *et al*, 2013; Zenz *et al*, 2005). Interestingly, a regional loss of *JunB* expression is observed in human psoriatic epidermis (Guinea-Viniegra *et al*, 2014). A similar observation has been made for systemic lupus erythematosus (SLE) with cutaneous involvement (Pflegerl *et al*, 2009).

Embryonic deletion of both *Jun* and *JunB* within the epidermis leads to fatal cachexia of neonatal mice (Guinea-Viniegra *et al*, 2009; Zenz *et al*, 2005). Their deletion in adult mice via a tamoxifen (Tx)-inducible cre recombinase in keratin 5 expressing cells (*Jun*^*f/f*^ *JunB*^*f/f*^ *K5cre*^*ER*^ *=* DKO* mice) leads within 14 days after Tx treatment to a skin phenotype that is strongly reminiscent of human psoriasis (Zenz *et al*, 2005). DKO* mice present many psoriatic hallmarks, ranging from epidermal changes such as keratinocyte hyperproliferation, parakeratosis, and prominent rete ridge formation to epidermal and dermal immune infiltrates, excess of proinflammatory cytokines (Zenz *et al*, 2005) and hypervascularization (Schonthaler *et al*, 2009). Additionally, DKO* mice exhibit molecular parallels to human psoriasis, specifically a similar global protein expression pattern (Schonthaler *et al*, 2013), complement activation (Schonthaler *et al*, 2013), and increased TNF-α shedding (Zenz *et al*, 2005).

In this study, we employed patient biopsies, an Imiquimod (Imi)-induced skin inflammation mouse model, and the DKO* mice to investigate the function of LCs and pDCs in psoriasis. We show that LC numbers were severely diminished within human psoriatic plaques, while pDC numbers were increased. In order to investigate the consequences of LC and pDC absence during defined phases of psoriatic inflammation, we employed DKO* mice bred to either *Langerin-DTR* (*LanDTR*) mice (Kissenpfennig *et al*, 2005), or to *BDCA2-DTR* mice (Swiecki *et al*, 2010), in which LCs or pDCs could be inducibly depleted by injection of DT, respectively. We found that depletion of pDCs prior to disease initiation attenuated disease development in the DKO* model, whereas their depletion during fully developed psoriasis-like inflammation had no effect. Conversely, LCs were not essential during the initiation of the phenotype, but their depletion during ongoing disease exacerbated skin inflammation. Our findings demonstrate that pDCs, which infiltrate during the early disease phase, are important instigators of psoriasis-like disease, while LCs serve to protect immune homeostasis in established inflammation.

## Results

### pDCs and LCs in human psoriatic lesions

**Figure 1:**
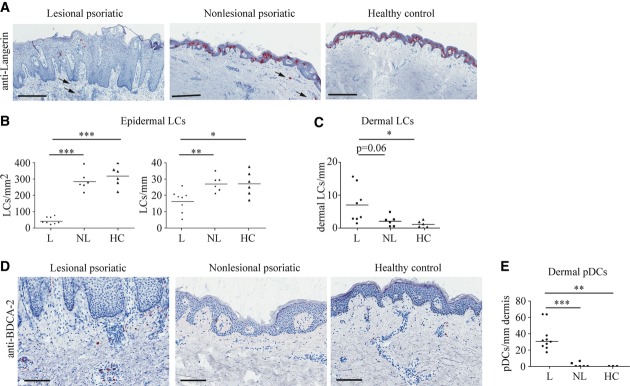
pDCs and LCs in human psoriatic lesions A Representative images of sections of psoriatic lesional (L) and non-lesional (NL), as well as healthy (H) donor skin stained with an antibody to Langerin. Scale bars represent 400 μm. Arrows indicate LCs in the dermis. B, C Numbers of (B) epidermal LCs per mm^2^ or per mm epidermis and (C) dermal LCs measured per mm epidermis on two independent sites per sample (*n* = 6–8). D Representative images of human skin sections stained with an antibody to BDCA-2. Scale bars indicate 150 μm. E Number of pDCs per mm dermis counted on 2 independent sites per sample (*n* = 3–10). Data information: Data were analyzed using unpaired Student's *t*-test (*P < 0.05, **P < 0.01, ***P < 0.001). *P*-values for this figure are available in Supplementary Table S3. Source data are available online for this figure.

So far, reports on LC numbers in psoriatic lesions have been inconsistent (Romani *et al*, 2012). Therefore, we carefully assessed LC numbers within skin biopsies from psoriatic lesions or non-lesional skin 2 cm distant from the lesional margin, as well as within skin obtained from age-matched healthy donors. While LC numbers within healthy skin and non-lesional sites of psoriatic patients were comparable, lesional skin revealed a significant reduction of LC numbers, when measured relative to epidermal area as well as to epidermal length (Fig 1A and B). Concomitantly, increased numbers of LCs were present in lesional dermis (see arrows in Fig 1A), suggesting that LC loss in psoriasis might be due to the enhanced migration of LCs through the dermis (Fig 1C).

Contrary to what was observed for LCs, the number of pDCs was significantly increased within lesional skin (Fig 1D and E), where pDCs accumulated predominantly in the papillary dermis, in line with what was previously reported for human psoriasis (Wollenberg *et al*, 2002). In contrast, in non-lesional skin, only very low numbers of pDCs were present (Fig 1D and E). These results show that in human psoriatic lesions, the number of LCs is dramatically decreased, whereas the number of pDCs is increased.

### Distribution of DC subtypes in inflamed skin of DKO* mice

**Figure 2:**
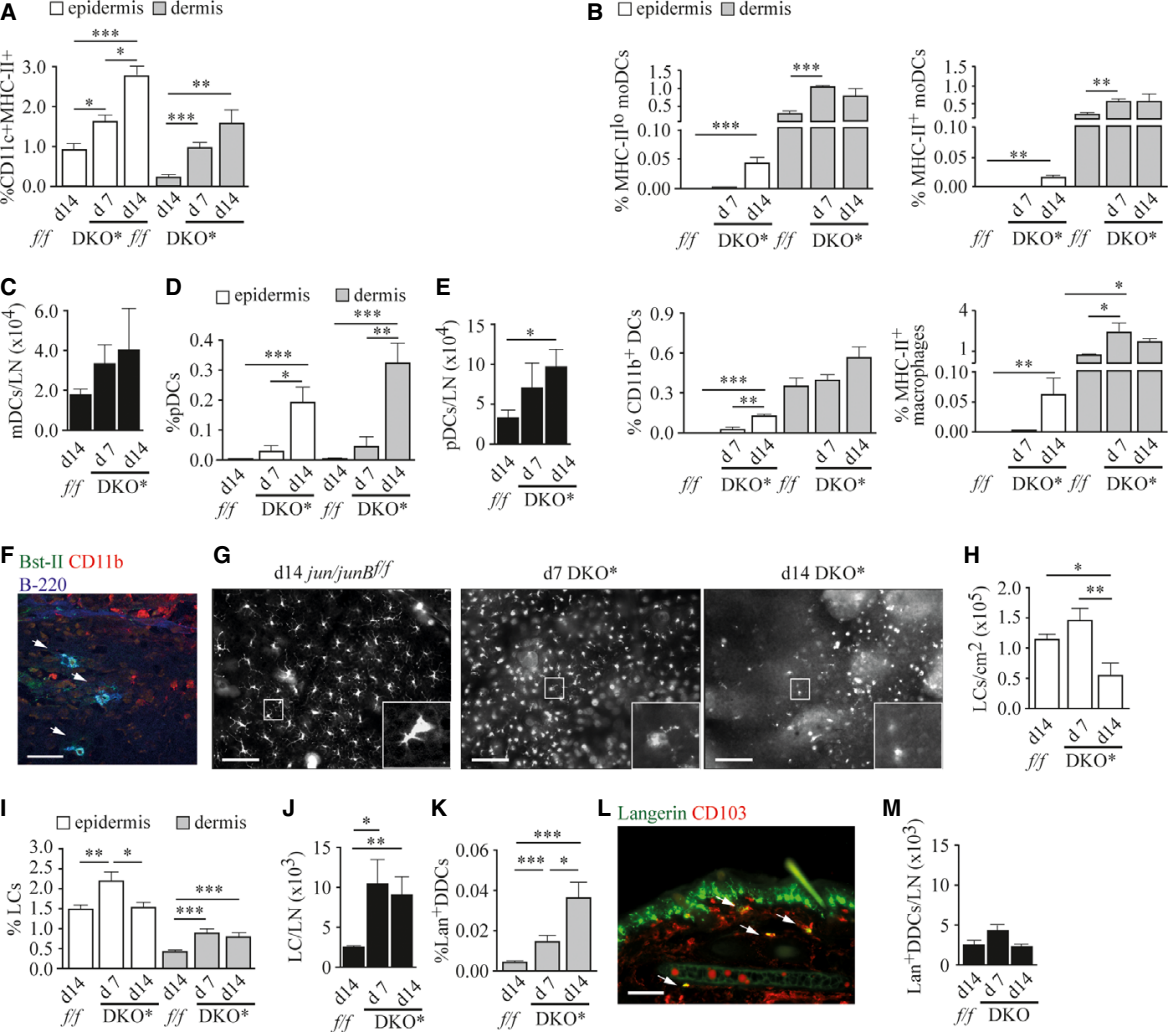
APC subtypes in inflamed skin of DKO* mice A, B Quantification of flow cytometric analysis of (A) ear epidermal and dermal APCs (*n* = 7–10), and (B) ear epidermal and dermal APCs after exclusion of LCs and Lan^+^ DDCs. Quantification of MHC-II^lo^ moDCs, MHC-II^+^ moDCs, CD11b^+^ DCs, and MHC-II^+^ macrophages (*n* = 3–4). C Quantification of migratory DCs (CD11c^+^MHC-II^hi^ cells) in auricular lymph nodes (*n* = 5–7). D, E Quantification of (D) ear epidermal and dermal pDCs (CD45^+^CD11c^+^Bst-II^+^B-220^+^CD11b^neg^ cells) (*n* = 5–8), and (E) pDCs (CD45^+^CD11c^+^Bst-II^+^B-220^+^CD11b^neg^ cells) in auricular lymph nodes (*n* = 6–9) of indicated mice 7–14 days after disease induction measured by flow cytometry. F Histological ear section stained for Bst-II (green), CD11b (red), and B-220 (blue). Nuclear staining: Hoechst (brown). Arrows indicate double positive (Bst-II/B-220) cells. Scale bar indicates 50 μm. G Representative images of epidermal ear sheets of indicated mice stained for Langerin. Scale bars indicate 100 μm. H Epidermal LC numbers counted on ear sheets at the indicated time points. At least three randomly chosen fields were counted for each sample (*n* = 9–10). I,J Quantification of (I) ear epidermal (CD45^+^Lan^+^ cells) and dermal (CD45^+^Lan^+^CD103^neg^ cells) LCs (*n* = 8–20), and (J) LCs in auricular lymph nodes (Lan^+^CD8^neg^CD11b^+^CD103^neg^) (*n* = 8–10). K Quantification of ear dermal Lan^+^ DDCs (CD45^+^Lan^+^CD103^+^ cells) (*n* = 8–15) of indicated mice 7–14 days after disease induction measured by flow cytometry. L Representative image of an immunofluorescent staining of an ear section of a day 14 DKO* mouse stained for Langerin (green) and CD103 (red). Arrows indicate double-positive cells, scale bar indicates 100 μm. M Quantification of Lan^+^ DDCs (Langerin^+^CD8^neg^CD11b^lo_to_+^CD103^+^) in auricular lymph nodes of indicated mice (*n* = 8–10) by flow cytometry. Data information: Flow cytometric quantifications are depicted as percentage of live cells. Data represent mean ± SEM. Data were analyzed using unpaired Student's *t*-test (*P < 0.05, **P < 0.01, ***P < 0.001). *P*-values for this figure are available in Supplementary Table S3. Source data are available online for this figure.

To mechanistically investigate the functional consequences of LC and pDC changes in psoriatic lesions, we employed the DKO* mice as a model for psoriasis. These mice develop a psoriasis-like skin disease upon tamoxifen (Tx)-induced deletion of *Ju*n and *JunB* in the epidermis with K5-cre^ER^. The psoriatic phenotype is fully developed after 14 days (d) and reproduces many major hallmarks of psoriasis (Zenz *et al*, 2005). On d7 after disease induction, ears and tails of DKO* mice exhibited mild erythema and scaling (Supplementary Fig S1A). Between d7 and d14, massive epidermal thickening as well as neutrophil and monocyte infiltration was observed that persisted when mice were continuously treated with Tx (Supplementary Fig S1A and B, and data not shown). No skin phenotype could be detected in Tx-treated control and *Jun/JunB*^*f/f*^ mice (Supplementary Fig S1A). The skin contains a wide spectrum of myeloid cells, which includes DCs, monocytes, and macrophages, which have been well characterized in a recent study (Tamoutounour *et al*, 2013). Flow cytometric analysis of this subset revealed a progressive increase in the frequency of MHC-II^+^CD11c^+^ cells in both epidermis and dermis of DKO* mice (Fig 2A, Supplementary Fig S1C). A more detailed analysis revealed that the increase in this population at d7 after disease induction is due to infiltration of several types of myeloid cells, including monocyte-derived DCs (moDCs) of the MHC-II^lo^ and MHC-II^+^ subsets (Fig 2B), that have been suggested to carry out specialized functions in inflammation (Villadangos & Schnorrer, 2007). In parallel, the frequency of MHC-II^+^ macrophages was increased in the dermis at d7, whereas CD11b^+^ DCs were not significantly altered compared to controls (Fig 2B). This demonstrates that in DKO* mice, the dermal myeloid cell composition is already considerably changed in an early phase of psoriatic inflammation. A similar pattern of myeloid cells can be found in the epidermis at d14, when cutaneous inflammation was already obvious (Fig 2B). As skin inflammation progressed, the fraction of migratory DCs also increased in auricular lymph nodes (Fig 2C, Supplementary Fig S1D). Since previous work had suggested a role for pDCs in psoriasis (Nestle *et al*, 2005), we analyzed their frequencies in DKO* mice. While pDCs were absent from the skin of *Jun/JunB*^*f/f*^ mice, they were significantly increased within the epidermis and dermis of d14 DKO* mice (Fig 2D, Supplementary Fig S1E), strongly resembling human disease (Fig 1D and E) (Nestle *et al*, 2005). Moreover, pDCs accumulated over the disease course within auricular lymph nodes (Fig 2E). pDCs present in psoriatic skin were mostly localized within the papillary dermis (Fig 2F), but were also present within the reticular dermis and the epidermis (data not shown), as has been described for psoriatic patients (Wollenberg *et al*, 2002).

We next investigated LC numbers on whole-mount epidermal ear sheets and within skin cell suspensions of DKO* mice. Epidermal LC numbers had significantly increased by d7, but were markedly reduced at d14 (Fig 2G–I, Supplementary Fig S1F). This reduction was consistent with our results in psoriatic patients (Fig 1A and B). Of note, while LCs were reduced at d14, epidermal Lan^neg^CD11c^+^MHC-II^+^ APC frequency was increased at d7 and further increased until d14 (Supplementary Fig S1G). Sections of inflamed ears revealed that LCs were confined to the suprabasal epidermis and had elongated dendrites protruding toward upper epidermal layers (Supplementary Fig S1H). In the dermis and auricular lymph nodes, LCs were present with increased frequency both at d7 and d14, indicating enhanced migration of LCs in psoriatic disease, likely explaining the observed epidermal LC loss (Fig 2I and J, Supplementary Fig S1I and J). No significant increase in the frequency of activated caspase-3 positive LCs could be detected in DKO* mice at both time points (Supplementary Fig S1K), excluding apoptosis as a cause for LC loss. Additionally, several DC activation markers, including CD40, CD80, CD86, and DEC-205, were upregulated on LCs in the epidermis and in lymph nodes of DKO* mice, which is in line with enhanced migratory activity of LCs in DKO* mice (Supplementary Fig S1L).

We also analyzed the behavior of another pro-inflammatory DC subset, Lan^+^ DDCs, during disease progression. Parallel to disease initiation, the CD103^+^ Lan^+^ DDCs infiltrated the dermis of affected skin of DKO* mice (Fig 2K and L). In contrast to LCs, Lan^+^ DDC numbers were not increased within auricular lymph nodes of DKO* mice (Fig 2M). Together, these results demonstrate that distinct DC subpopulations undergo spatiotemporal reorganization during psoriasis-like disease development and progression, similar to the situation in humans. LCs were increased in the epidermis during the initiation phase, whereas their frequency decreased with disease progression. This was paralleled by increased emigration of LCs to the dermis and lymph nodes. Moreover, ‘Inflammatory-type’ DCs, such as pDCs and the CD103^+^ Lan^+^ DDCs, were increased in the dermis of psoriatic mice.

### pDCs are necessary for the induction of psoriatic disease, but dispensable for its maintenance

**Figure 3:**
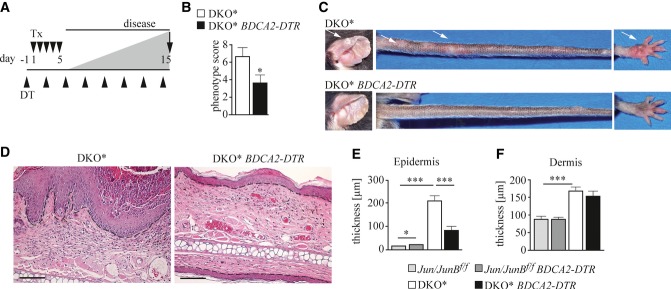
pDCs are necessary for the induction of psoriatic disease A Mice were injected with DT (▴) 1 day before inducing disease by five daily consecutive injections of Tx (▾) and analyzed on day 15 after disease induction. B Mean psoriatic phenotype score (see Materials and Methods for details) of the indicated mice was determined on day 15 (*n* = 12–13). C Representative image of affected body parts of indicated mice on day 15. Arrows indicate lesions. D Representative H&E staining of ear sections of indicated mice. Scale bars indicate 100 μm. Dashed line indicates epidermal–dermal junction. E, F Histogram showing (E) epidermal and (F) dermal thickness of mice of the indicated genotype. Ten randomly chosen fields of 3–4 independent images per mouse were analyzed (*n* = 9–12). Magnification 4×. *Jun/JunB*^*f/f*^: light gray, *Jun/JunB*^*f/f*^ *BDCA2-DTR*: dark gray, DKO*: white, and DKO* *BDCA2-DTR*: black. Data information: Data represent mean ± SEM. Data were analyzed using Mann–Whitney *U*-test (*P < 0.05, **P < 0.01, ***P < 0.001). *P*-values for this figure are available in Supplementary Table S3. Source data are available online for this figure.

In order to investigate pDC function in psoriasis-like disease, we crossed DKO* mice with *BDCA2-DTR* mice, that can be selectively depleted of pDCs by application of DT (Swiecki *et al*, 2010). This strategy allowed us to eliminate pDCs present in the spleen and dermis (Supplementary Fig S2A–D). To compare disease severity between individuals and time points, we estimated the disease phenotype using a blinded scoring system based on the observed redness, scaliness, and plaque size and density (see Materials and Methods for detailed description). When pDCs were depleted before disease initiation (Fig 3A), the average disease score of DKO* mice was significantly reduced on d15 after disease induction, when most of the pDC-sufficient mice had developed a pronounced phenotype (Fig 3B). pDC-depleted DKO* mice had visibly less inflamed skin when compared to pDC-sufficient DKO* mice (Fig 3C, arrows indicating typical psoriatic lesions). Concomitantly, epidermal thickening was reduced in pDC-depleted DKO* mice, while dermal thickness were not altered (Fig 3D–F). However, pDC depletion at d14 when lesions were already pronounced (Supplementary Fig S2E) had no impact on disease progression (Supplementary Fig S2F and G), or epidermal and dermal thickening (Supplementary Fig S2H, I and J). These data demonstrate that pDCs play an essential role in psoriasis initiation (Nestle *et al*, 2005).

Next, we reexamined our findings using another widely used mouse model of psoriasis-like disease, which is based on the topical application of the TLR7 agonist Imiquimod (Imi) (van der Fits *et al*, 2009). Thus, *BDCA2-DTR* mice were treated with either PBS or DT 1 day before Imi application (Supplementary Fig S2K). We found that depletion of pDCs prior to Imi treatment did not influence skin inflammation induced by 6 daily consecutive Imi applications (Supplementary Fig S2L and M), confirming recent findings (Wohn *et al*, 2013) and indicating that the two models (DKO* and Imi) exhibit molecular differences.

### The psoriatic phenotype of DKO* mice is exacerbated when Lan^+^ APCs are depleted during chronic psoriasis-like disease

**Figure 4:**
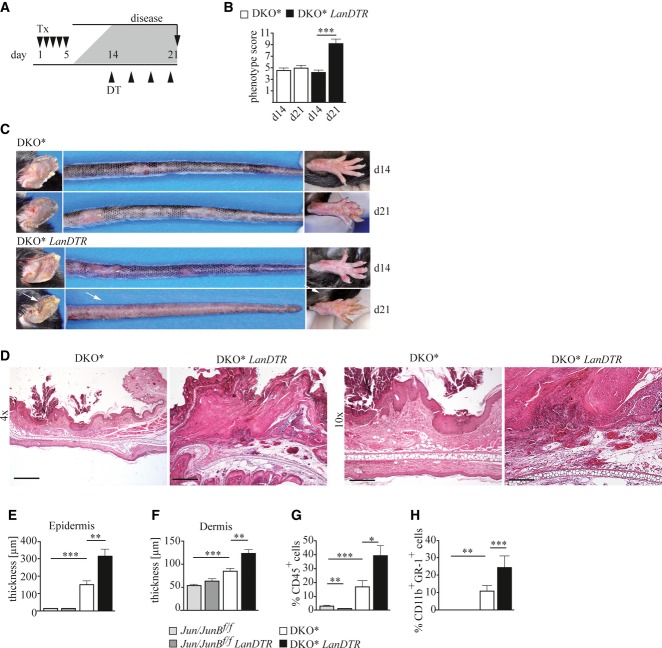
The psoriatic phenotype of DKO* mice is exacerbated when Lan^+^ APCs are depleted during chronic disease A Psoriatic disease was induced by five daily consecutive injections of Tx (▾), and Lan^+^ APCs were depleted by injection of DT (▴) at day 14 when psoriasis had developed (injections every third day). Mice were euthanized on day 21. B Mean psoriatic phenotype score of the indicated mice was determined on day 14 and day 21 after disease induction (*n* = 39–41). C Representative images of affected body parts of DKO* and DKO* *LanDTR* mice on day 14 and day 21 are shown. Arrows indicate sites of aggravated inflammation after Lan^+^ APC depletion. D Representative H&E staining of ear sections of indicated mice on day 21. Scale bars represent 500 μm (magnification 4×) and 200 μm (magnification 10×). E, F Histogram showing (E) epidermal and (F) dermal thickness of skin of mice of the indicated genotype. Ten randomly chosen fields of 3–4 independent images per mouse were analyzed (*n* = 9–19), magnification 4×. G, H Quantification of flow cytometric analysis showing (G) ear and tail epidermal immune cells (CD45^+^ cells), and (H) epidermal neutrophils/monocytes (CD45^+^Gr-1^+^CD11b^+^ cells) of indicated mice (*n* = 8–12). *Jun/JunB*^*f/f*^*:* light gray*, Jun/JunB*^*f/f*^ *LanDTR*: dark gray, DKO*: white, and DKO* *LanDTR*: black. Data information: Flow cytometric quantifications are depicted as percentage of live cells. Data represent mean ± SEM. Data for (B), (G), and (H) were analyzed using Wilcoxon signed-rank test, and for (E) and (F), using Mann–Whitney *U*-test (*P < 0.05, **P < 0.01, ***P < 0.001). *P*-values for this figure are available in Supplementary Table S3. Source data are available online for this figure.

To investigate the function of LCs in psoriasis, we crossed DKO* mice with *LanDTR* mice, in which DT injection ablates all Lan^+^ APCs including epidermal LCs, and Lan^+^ DDCs which are found in the dermis (Kissenpfennig *et al*, 2005) (Supplementary Fig S3A–D). In control *Jun/JunB*^*f/f*^ mice, depletion of Lan^+^ APCs did not affect skin homeostasis. To determine whether Lan^+^ APCs play a role in the induction of psoriatic disease, we depleted Lan^+^ APCs starting 1 day before disease induction (Supplementary Fig S3E). Under these conditions, mice depleted of Lan^+^ APCs displayed a similar psoriatic phenotype as their Lan^+^ APC-sufficient littermates (Supplementary Fig S3F–J). In contrast, when Lan^+^ APCs were depleted during the chronic phase of psoriasis-like skin disease on d14 (Fig 4A), we observed severe aggravation of the inflammation, whereas in Lan^+^ APC-sufficient DKO* mice, the psoriatic phenotype remained relatively constant (Fig 4B). Disease aggravation was characterized by a massive increase in erythema, as well as in density and severity of psoriatic plaques (Fig 4C, Supplementary Fig S3K). Furthermore, increased epidermal hyperplasia as well as epidermal and dermal inflammation could be detected (Fig 4D). As a result, both epidermal and dermal thickening were significantly increased in Lan^+^ APC-depleted compared to Lan^+^ APC-sufficient DKO* mice (Fig 4E and F).

In psoriasis, characteristic immune cell subtypes are present in the epidermis, as aggregates of neutrophils (Munro's microabscesses), as well as inflammatory DCs (Lowes *et al*, 2005) and T cells (Schon & Boehncke, 2005). Flow cytometric analysis revealed that the epidermis of DKO* mice contained a massive infiltrate consisting of cells of hematopoietic origin (CD45^+^) that was significantly increased in Lan^+^ APC-depleted mice (Fig 4G). The largest epidermal immune cell fraction was represented by myeloid cells consisting of a mixture of CD11b^+^ Gr-1^high^ and CD11b^+^ Gr-1^int^ cells, which were considerably increased in Lan^+^ APC-depleted DKO* mice (Fig 4H). Furthermore, the frequency of intraepidermal Langerin-negative (Lan^neg^) infiltrating CD11c^+^ MHC-II^+^ APCs was significantly increased in Lan^+^ APC-depleted compared to Lan^+^ APC-sufficient DKO* mice (Supplementary Fig S3L). In addition, epidermal T-cell frequencies were significantly higher in psoriatic DKO* mice and had a tendency to be further increased upon LC depletion (Supplementary Fig S3M). The increase in T-cell frequencies in DKO* mice was most likely attributed to higher numbers of TCRαβ^+^ T cells, mostly of the CD4^+^ but also the CD8^+^ expressing subset, whereas the frequency of γδ T cells was similar between control and DKO* mice (Supplementary Fig S3N). Since LCs had anti-inflammatory function in DKO* mice, we investigated whether LC frequency and fate were altered by pDC depletion. Neither epidermal nor dermal LC numbers were significantly changed between pDC-sufficient and pDC-depleted DKO* mice, excluding a counterregulation of LC numbers through pDCs (Supplementary Fig S3O and P). Taken together, these results demonstrate that ablation of Lan^+^ APCs in the skin during the chronic phase of psoriatic disease leads to increased psoriatic inflammation, suggesting that Lan^+^ APCs have the capacity to downregulate chronic inflammation in this model.

To exclude that the aggravation of the psoriatic phenotype was due to DT-induced death of Lan^+^ APCs per se rather than their absence, we depleted Lan^+^ APCs in the Imi model of skin inflammation. Depletion of Lan^+^ APCs before Imi-induced skin inflammation (induced by 6 daily consecutive treatments) did not change its severity as measured on d7 (Supplementary Fig S4A–C). Also, depletion of Lan^+^ APCs before Imi application every other day for 14 days (Drobits *et al*, 2012; Palamara *et al*, 2004) did not impact on disease severity (Supplementary Fig S4D–F). Importantly, depletion of Lan^+^ APCs during the propagation phase at d5 of Imi treatment, when skin was visibly inflamed, did also not aggravate the observed phenotype (Supplementary Fig S4G–I), demonstrating that Lan^+^ APC depletion within inflamed skin per se does not result in an enhanced inflammatory reaction.

### LCs, but not Lan^+^ DDCs, counteract psoriatic inflammation of DKO* mice

**Figure 5:**
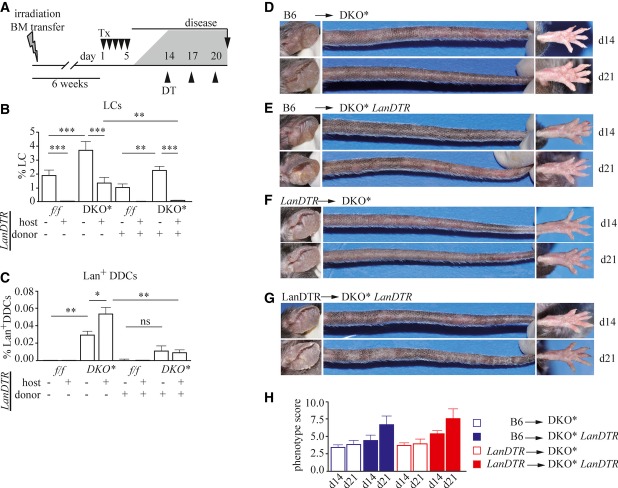
Anti-inflammatory effects are mediated by LCs, but not by Lan^+^ DDCs, in psoriasis A DKO* *LanDTR* mice were irradiated and intravenously reconstituted with bone marrow isolated from either C57BL/6J (B6) or *LanDTR* mice expressing CD45.1. Psoriatic inflammation was induced by Tx (▾) injection. On day 14, indicated cell types were depleted by application of DT (▴). Mice were euthanized 7 days after the first DT injection. B, C Quantification of (B) epidermal LCs (Lan^+^CD45^+^ cells) (*n* = 4–7), and (C) dermal Lan^+^ DDCs (CD45^+^CD11c^+^MHC-II^+^Lan^+^CD103^+^ cells) (*n* = 4–7) by flow cytometry. D–G Representative pictures of affected body parts of chimeric DKO* mice: (D) DKO* mice carrying B6 bone marrow (B6 → DKO*), (E) DKO* *LanDTR* mice carrying B6 bone marrow (B6 → DKO* *LanDTR*), (F) DKO* mice carrying *LanDTR* bone marrow (*LanDTR* → DKO*), and (G) DKO* *LanDTR* mice carrying *LanDTR* bone marrow (*LanDTR* → DKO* *LanDTR*). H Mean psoriatic phenotype score of the indicated mice was determined on day 14 and day 21 after disease induction (*n* = 6–8). Data information: Flow cytometric quantifications are depicted as percentage of live cells. Data represent mean ± SEM. Data for (B) and (C) were analyzed using unpaired Student's *t*-test (*P < 0.05, **P < 0.01, ***P < 0.001). *P*-values for this figure are available in Supplementary Table S3. Source data are available online for this figure.

Since injection of DT efficiently eliminated not only epidermal LCs, but also other Lan^+^ APCs, including Lan^+^ DDCs, we next addressed which of these cell types is responsible for the suppression of the psoriatic phenotype seen in DKO* *LanDTR* mice. For this purpose, a series of bone marrow chimeric mice were generated, in which either LCs, Lan^+^ DDCs, or both could selectively be depleted. After lethal gamma irradiation followed by transplantation of a donor bone marrow, LCs remain of host origin, whereas most immune cells are replaced from the donor bone marrow (Merad *et al*, 2008). We irradiated DKO* or DKO* *LanDTR* hosts and reconstituted them with bone marrow of control C57BL/6J (B6) or *LanDTR* (*LanDTR*) mice expressing CD45.1, a naturally occurring polymorphism of the CD45 antigen, as a marker. After reconstitution, psoriatic disease was induced, and DT was injected 14 days after disease induction (Fig 5A). After DT injection, DKO* mice reconstituted with control B6 bone marrow (B6 → DKO*) had normal frequencies of LCs and Lan^+^ DDCs, and disease remained constant (Fig 5B–D and H). In DKO* *LanDTR* mice reconstituted with a *LanDTR* bone marrow (*LanDTR* → DKO* *LanDTR*), in which both LCs and Lan^+^ DDC were depleted, psoriasis-like inflammation was exacerbated upon DT injection (Fig 5B, C, G, and H) similarly to what was observed with DKO* *LanDTR* mice (Fig 4B and C). Also, DKO* mice expressing LanDTR engrafted with B6 bone marrow (B6 → DKO* *LanDTR*), in which DT application depleted LCs, but not Lan^+^ DDCs, exhibited a more severe phenotype after DT application (Fig 5B, C, E, and H). In contrast, depletion of only Lan^+^DDCs, but not LCs in DKO* mice carrying bone marrow isolated from *LanDTR* mice (*LanDTR* → DKO*), did not have a significant impact on disease progression (Fig 5B, C, F, and H). These results demonstrate that LCs exert an attenuating function on psoriatic skin inflammation, whereas Lan^+^ DDCs and other Langerin-expressing DC subsets were dispensable for the progression of the psoriatic phenotype.

**Figure 6:**
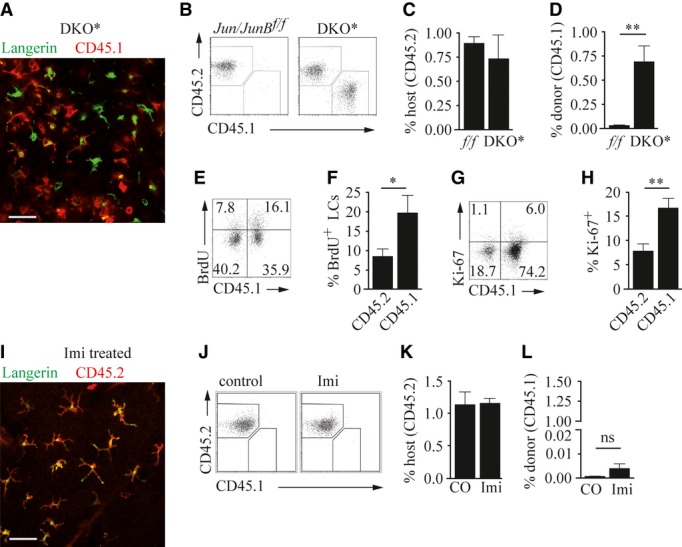
LCs in chimeric DKO* mice originate from the bone marrow A–H Lethally irradiated DKO* mice were reconstituted with CD45.1-expressing bone marrow cells, before psoriatic disease was induced. Mice were analyzed on day 9 after disease induction. (A) Representative image showing epidermal ear sheet of a DKO* mouse stained for Langerin (green) and CD45.1 (red). Scale bar indicates 100 μm. (B) Flow cytometric analysis of ear and tail epidermal LCs (CD45^+^Lan^+^ cells) that express CD45.2 (host-derived) or CD45.1 (donor-derived), and quantification of (C) host-derived LCs (CD45^+^Lan^+^ cells) and (D) donor-derived LCs (CD45^+^Lan^+^ cells) (*n* = 6) of indicated mice. (E) Flow cytometric analysis showing BrdU incorporation in host- or donor-derived ear and tail epidermal LCs (CD45^+^Langerin^+^ cells) and (F) quantification of BrdU^+^ LCs of host- or donor-derived origin (*n* = 16). (G) Flow cytometric analysis showing Ki-67 expression by host- or donor-derived ear and tail epidermal LCs (CD45^+^Langerin^+^ cells) and (H) quantification of Ki-67^+^ LCs of host- or donor-derived origin (*n* = 16). I–L C57BL/6J mice were reconstituted with CD45.1-expressing syngeneic bone marrow, and mouse ears were treated daily with Imiquimod (Imi) for 6 days and analyzed on day 8. (I) Representative image of an epidermal ear sheet of a DKO* mouse stained for Langerin (green) and CD45.2 (red). (J) Flow cytometric analysis showing host (CD45.2^+^) and donor (CD45.1^+^) contribution to ear and tail epidermal LCs (CD45^+^Lan^+^ cells). (K) Percentage of host-derived (CD45.2^+^) LCs (*n* = 2–4) or (L) donor-derived (CD45.1^+^) LCs of total epidermal cells of the indicated mice (*n* = 2–4). Data information: Flow cytometric quantifications are depicted as percentage of live cells. Data were analyzed using Mann–Whitney *U*-test (*P < 0.05, **P < 0.01). Scale bars in (A) and (I) indicate 100 μm. *P*-values for this figure are available in Supplementary Table S3. Source data are available online for this figure.

It has been shown that application of experimental conditions involving severe skin inflammation leads to repopulation of LCs by bone marrow-derived precursors (Ginhoux *et al*, 2006; Nagao *et al*, 2012; Sere *et al*, 2012). In DKO* mice, we noticed that LCs were in part derived from the bone marrow. To determine whether LCs repopulate the epidermis in psoriatic inflammation, and whether this leads to recruitment of a bone marrow-derived progenitor, we lethally irradiated CD45.2 expressing DKO* mice and engrafted them with bone marrow isolated from C57BL/6J mice expressing CD45.1. After reconstitution, we induced psoriatic inflammation. Nine days after disease induction, we found host- as well as donor-derived LCs within the epidermis (Fig 6A and B). Interestingly, the frequency of host-derived LCs remained relatively constant in DKO* mice (Fig 6C). Furthermore, while in the epidermis of control mice, only 5% of LCs were of donor origin, LCs in DKO* mice were on average 40% of donor origin (Fig 6D). In line with this, donor-derived LCs exhibited a higher rate of BrdU incorporation (Fig 6E and F) and higher Ki-67 expression levels (Fig 6G, H) than host-derived LCs found in psoriasis-like disease. In contrast, topical Imi treatment did not result in substantial recruitment of bone marrow-derived LCs (Fig 6I–L). These results demonstrate that in psoriasis-like disease of DKO* mice, a considerable fraction of LCs are derived from the bone marrow. Additionally, bone marrow-born LCs exhibited higher proliferative activity and also proliferated more potently *in situ* within the epidermis when compared to host-derived LCs, which suggests a progressive turnover of the resident LC compartment by bone marrow-derived LCs in psoriasis-like disease.

### Inhibition of the IL-23 pathway ameliorates psoriasis

**Figure 7:**
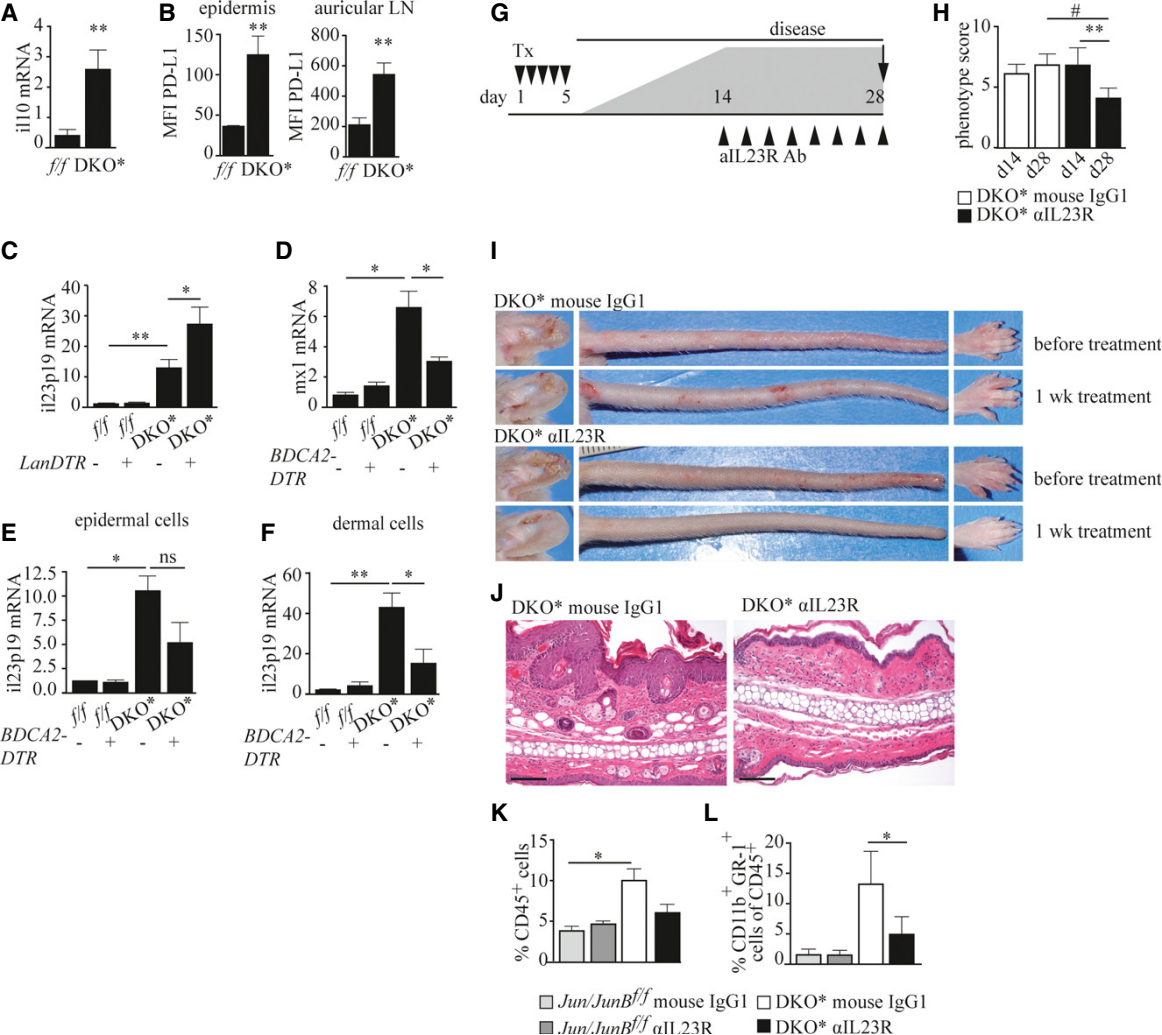
pDCs and LCs influence disease severity via IL-23 A Relative Il10 mRNA expression in isolated LCs of indicated mice (*n* = 7–8). B PD-L1 expression on epidermal and auricular lymph node LCs (*n* = 3). C Relative Il23 mRNA expression in epidermal cells of indicated mice treated as described in Fig 4A (*n* = 4–6). D Relative Mx1 mRNA expression (*n* = 3–7). E, F Relative Il23 mRNA expression in (E) epidermal (*n* = 3–7) and (F) dermal cells (*n* = 3) of indicated mice treated as described in Fig 3A. G Psoriatic disease was induced by five daily consecutive injections of Tx (▾), and on day 14, when psoriatic disease had developed, mice were treated with an inhibitory anti-IL-23R antibody or an isotype control every other day (▴), and euthanized on day 28. H Psoriatic inflammation scored at day 14 and day 28 of the indicated mice (*n* = 9–14). I Representative images of affected body parts of DKO* mice before and after treatment with anti-IL-23R or isotype control antibody. J Representative H&E-stained ear sections of indicated mice on day 28. Scale bars represent 100 μm. K, L Quantification of (K) dermal immune cells (CD45^+^) (*n* = 3–4), and (L) dermal monocytes/neutrophils (Gr-1^+^CD11b^hi^ cells) within CD45^+^ dermal cells of indicated mice (*n* = 5–9) measured by flow cytometry. Data information: Flow cytometric quantification in (K) is depicted as percentage of live cells. Data represent mean ± SEM. Data for (A–F) and (K) were analyzed using unpaired Student's *t*-test, and for (H) and (L), using Wilcoxon signed-rank test (*P < 0.05, **P < 0.01) and for (H), additionally using Mann–Whitney *U*-test (^#^*P *< 0.05). *P*-values for this figure are available in Supplementary Table S3. Source data are available online for this figure.

We next investigated the mechanism by which LCs ameliorate and pDCs induce psoriatic inflammation. It has previously been shown that LC-derived IL-10 can mediate tolerance in response to UVB and in a skin graft model (Yoshiki *et al*, 2010, 2009). Moreover, PD-L expression on mature LCs has been associated with reduced T-cell activation (Pena-Cruz *et al*, 2010). We therefore examined the expression of these regulatory mediators in LCs isolated from DKO* mice and found that LCs from mice with psoriasis-like skin inflammation produced substantially higher levels of IL-10 than those derived from *jun/junB*^*f/f*^ mice (Fig 7A, Supplementary Fig S5A). Additionally, LCs isolated from the skin and skin-draining LN of DKO* mice showed strongly increased surface expression of PD-L1 (Fig 7B). Thus, in the absence of LCs, the lack of these regulatory signals might result in increased inflammation in the skin of DKO* mice. LC depletion was not associated with changes in the numbers of regulatory T cells (T_reg_) in the skin or in skin-draining lymph nodes (Supplementary Fig S5B–D) excluding that reduced numbers of T_reg_ were responsible for disease aggravation. IL-23, one of the key cytokines promoting the development of psoriasis, was significantly upregulated in psoriatic epidermis of DKO* mice and depletion of LCs during the chronic disease phase resulted in further elevation of this inflammatory cytokine (Fig 7C). It was previously reported that in DKO* mice, both IL-17f and IL-17a are highly expressed (Schonthaler *et al*, 2013). We also found that epidermal IL-17f and IL-22 expression were increased in DKO* mice. These cytokines were, however, not significantly altered in the absence of LCs (Supplementary Fig S5F and G).

In psoriasis, pDCs are the most potent producers of type I interferons (IFNs) (Nestle *et al*, 2005). Accordingly, in DKO* mice, epidermal expression of the interferon-responsive gene *Mx1* was strongly upregulated (Fig 7D). Depletion of pDCs prior to disease induction resulted in significant reduction of Mx1 expression suggesting that reduced type-I-IFN signaling might contribute to disease amelioration (Fig 7D). Furthermore, in the absence of pDCs, the levels of IL-23 were significantly reduced in the dermis of DKO* mice (Fig 7E and F), whereas IL-17f and IL-22 levels were not significantly altered (Supplementary Fig S5H and I). Interestingly, intracellular FACS staining revealed that high levels of IL-17 were likely not produced by γδ T cells, but rather by a TCR γδ^neg^ subset, most likely representing αβ T cells (Supplementary Fig S5M). Accordingly, only γδ^neg^ T cells but not γδ T cells were increased in DKO* mice, and their frequency did not significantly change after pDC depletion (Supplementary Fig S5J–L). To determine whether this differential regulation of IL-23 expression in the skin could explain the distinct effects seen in the absence of selected DC subtypes, we treated DKO* mice with a blocking monoclonal antibody directed against the IL-23R. Psoriatic mice treated with the anti-IL-23R antibody for 2 weeks, starting from d14 after disease induction showed a markedly ameliorated phenotype, while the phenotypes of mice treated with isotype control antibody remained constant (Fig 7G–J). IL-23R blockage was associated with reduced psoriatic hallmarks (Fig 7J), as well as reduced amounts of intradermal immune cells (Fig 7K and L). These results demonstrate that LCs have an anti-inflammatory role during active psoriatic disease likely via the production of IL-10 and suppression of IL-23 production, while pDCs exert an instigator function during disease initiation by potentiating the IL-23 axis (Supplementary Fig S6).

## Discussion

The contribution of the distinct DC subsets that are present in the skin of psoriatic patients is only poorly understood. Recently, two studies suggested that a dermal DC subset may be involved in psoriasis initiation via the production of IL-23 (Wohn *et al*, 2013), a key cytokine that mediates expansion of IL-17- and IL-22-producing cells, promoting important events in psoriasis pathology, including neutrophil infiltration and epidermal thickening (Di Cesare *et al*, 2009). In the present study, we report that two other skin DC subtypes, pDCs and LCs, contribute selectively to distinct stages, initiation and propagation, of the inflammatory process in the skin according the model shown in Supplementary Fig S6.

pDCs were shown previously to be abundantly present within both lesional and non-lesional skin of psoriatic patients (Nestle *et al*, 2005). In contrast, we found significant numbers of pDCs only in psoriatic skin, with low numbers in non-lesional skin, at 2 cm distant from the adjacent lesion. Different to our investigation, the previous study analyzed skin 0.5 cm distant from the lesion, which might still represent an activated skin area in psoriasis (Nestle *et al*, 2005). In a mouse model of xenotransplanted human non-lesional skin of psoriatic patients, injection of anti-BDCA2 antibodies, which block pDC-specific IFN-α secretion, prevented the development of psoriatic lesions (Nestle *et al*, 2005). We also found that the presence of pDCs was necessary only for the initiation of psoriatic disease in DKO* mice since their depletion attenuated the psoriatic phenotype. pDCs were dispensable for maintenance of chronic inflammation, which might explain the inefficiency of anti-IFNα-based therapies in psoriatic patients (Bissonnette *et al*, 2010). In a second mouse model of skin inflammation that is based on the topical application of the synthetic TLR7 agonist Imiquimod, we and others (Wohn *et al*, 2013) have found that the development of skin inflammation was independent of pDCs. This discrepancy to the DKO* model might be due to the fact that Imiquimod induces only an acute and transient skin inflammation, thus mimicking only very early steps in psoriasis inflammation. In contrast, the DKO* psoriasis model exhibits chronic inflammation, which remains constant over a longer period of time, thereby likely modeling the human disease.

We found that LCs were reduced in lesions of psoriatic patients as well as in the DKO* psoriatic mouse model. In DKO* mice, the disappearance of LCs was independent of the presence of pDCs, since pDC depletion in DKO* mice did not affect epidermal LC frequencies. Other groups that reported a reduction of LC numbers in psoriatic skin found that LC numbers reverted back to normal levels when patients had successfully been treated (Romani *et al*, 2012). In another study, following skin trauma, a portion of LCs underwent mass emigration directly after the insult, while the majority of LCs did not emigrate upon further stimulation (Dearman *et al*, 2004). Similarly, in patients with early-onset psoriasis, LCs from non-lesional skin were unable to migrate in response to cytokine stimulation (Cumberbatch *et al*, 2006). Therefore, the differential migration capacities of LCs from psoriatic skin and the fact that only about 30% of LCs can be induced to emigrate, might reflect the existence of two types of LCs in humans. In support of this hypothesis are the observations that during inflammatory conditions, LCs can originate either from bone marrow-derived precursors or from preexisting epidermal LC precursors (Chorro *et al*, 2009; Elnekave *et al*, 2014; Ginhoux *et al*, 2006; Nagao *et al*, 2012). In DKO* mice, but not in Imi-treated mice, we found that depleted LCs were replaced from bone marrow rather than from skin-resident precursors, suggesting that two different types of LCs exist which might differently react to inflammatory conditions.

We found that elimination of LCs aggravated psoriasis-like inflammation. Lan^+^ DDCs, which have been shown to prime cutaneous adaptive immune responses in several instances (Romani *et al*, 2012), did not influence the chronic disease phase. LC depletion was associated with an increase in the frequency of epidermal immune cells known to be key mediators in psoriasis such as neutrophils, or Lan^neg^ APCs, that are efficient producers of TNF-α in psoriasis (Lowes *et al*, 2005). Multiple lines of evidence argue for a local or systemic tolerogenic role of LCs during inflammatory conditions such as UV irradiation (Yoshiki *et al*, 2010), allergic contact dermatitis (Gomez de Aguero *et al*, 2012), or infections (Kautz-Neu *et al*, 2011). In contrast, LCs are required for the efficient generation of immune responses in other situations, such as for antigen-specific T helper 17 (TH17) cells during fungal skin infections (Igyarto *et al*, 2011), and vaginal immunization (Hervouet *et al*, 2010). In DKO* mice, LCs within the epidermis as well as in lymph nodes exhibited higher levels of CD205, PD-L1, and CD86, which have been associated with DC-mediated generation of regulatory T cells (Bonifaz *et al*, 2002), peripheral T-cell tolerance (Salomon *et al*, 2001), and protection from spontaneous autoimmunity (Probst *et al*, 2005). PD-L1 can be upregulated in response to IL-10, which has also been implicated in the induction of peripheral tolerance. We detected increased IL-10 expression in LCs isolated from DKO* mice. IL-10 has been shown to negatively regulate the production of proinflammatory cytokines (D'Andrea *et al*, 1993; de Waal Malefyt *et al*, 1991). IL-10 production by DCs seems to be crucial for the establishment of tolerance after UV irradiation in the skin (Ghoreishi & Dutz, 2006). Psoriatic patient skin lacks IL-10 compared to healthy individuals, which is likely due to the severe reduction of LCs in psoriatic lesions (Nickoloff *et al*, 1994). Therefore, LCs may directly prevent psoriasis aggravation via IL-10 and PD-L1.

An increasing body of evidence points to a critical role for IL-23 signaling in the pathogenesis of psoriasis. We found that IL-23 was increased in the skin of DKO* mice and epidermal IL-23 levels seem to be antagonistically regulated by both types of DCs investigated. In the absence of LCs during the chronic phase of psoriatic inflammation, epidermal IL-23 levels increased, while absence of pDCs during psoriasis initiation led to a reduction of dermal IL-23 levels (Supplementary Fig S6). These changes in IL-23 did not affect the levels of IL-17 and IL-22. This is surprising, given the fact that IL-23 has been reported to mediate its effects by supporting a robust IL-17 response. However, two recent papers indicate that IL-23-driven pathology in both an asthma and a colitis model were independent of the presence of IL-17 (Izcue *et al*, 2008; Peng *et al*, 2010). Likewise, IL-23 stimulated the secretion of antimicrobial peptides in keratinocytes (Kanda & Watanabe, 2008), molecules that have been implicated in the instigation of psoriasis (Lowes *et al*, 2014).

Recently, in two studies, IL-23 production following stimulation with Imi was attributed to myeloid DCs such as conventional DCs (Wohn *et al*, 2013), CD103^+^ DC, and macrophages of the dermis (Riol-Blanco *et al*, 2014). The latter two populations are also present in high abundance in the early stage of psoriatic inflammation of DKO* mice. However, other groups have reported that IL-23 is produced by keratinocytes in psoriasis (Piskin *et al*, 2006). Therefore, it is possible that DCs and macrophages are involved in the early steps of psoriasis etiology, with keratinocytes taking over the production of IL-23 once the inflammatory cascade is fully pronounced.

We found that in DKO* mice that were treated with an antibody directed against the murine IL-23R, chronic psoriasis-like inflammation was significantly ameliorated. Successful therapy of psoriatic patients with antibodies targeting molecules within the IL-23 axis, such as Ustekinumab, an antibody against the p40 subunit shared by IL-12 and IL-23, has been established in clinical trials (Rustin, 2012). Furthermore, promising results in clinical trials were also obtained with an antibody targeting the specific p19 subunit of IL-23 (Alexander, 2013). However, antibody-based therapies are costly and come with a certain risk of side effects owing to systemic immunosuppression (Crow, 2012). Thus, strategies aimed at modulating the local composition of DC subtypes in psoriatic lesions might represent a novel approach for the treatment of psoriasis in the future.

## Materials and Methods

### Mice

Mice harboring loxP-flanked alleles of *Jun* and *JunB* and expressing *K5cre-ER*^*T*^ have been previously described (Zenz *et al*, 2005). *Jun*^*f/f*^*JunB*^*f/f*^ *K5cre-ER*^*T*^ mice (mixed background) were bred to *LanDTR* (Kissenpfennig *et al*, 2005) and *BDCA2-DTR* mice (Swiecki *et al*, 2010) (both of C57BL/6J background). To delete *Jun* and *JunB* and induce psoriasis-like disease, K5-cre^ER^ positive (DKO*) or negative (*Jun/JunB*^*f/f*^) mice were injected with 1 mg tamoxifen (Tx, Sigma-Aldrich) in an emulsion with sunflower seed oil (Sigma)/ethanol mixture (10:1) intraperitoneally on 5 consecutive days. Deletion of *Jun* and *JunB* was verified by PCR. Similarly, 300 ng of Diphtheria toxin (DT, List Biological Laboratories, in PBS) was injected intraperitoneally into experimental mice according to the schemes indicated in the figures. For LC depletion, DT was applied every third day, and for pDC depletion, every other day. LC and pDC depletion was > 90% as determined in the epidermis or the spleen, respectively. Mice were kept in the animal facility of the Medical University of Vienna in accordance with institutional policies and federal guidelines. Animal experiments were approved by the Animal Experimental Ethics Committee of the Medical University of Vienna and the Austrian Federal Ministry of Science and Research. (Animal license numbers: GZ 66.009/124-BrGT/2003; GZ 66.009/109-BrGT/2003; BMWF-66.009/0073-II/10b/2010 BMWF-66.009/0074-II/10b/2010; BMWFW-66.009/0200-WF/II/3b/2014; and BMWFW-66.009/0199-WF/II/3b/2014).

### Patient material, histology, and histomorphometry

Skin was obtained under an approved protocol (EK700/2009, Ethics Committee of the Medical University of Vienna), according to the Declaration of Helsinki. Patients suffering from chronic plaque-type psoriasis with a PASI > 10 that had undergone no systemic or topical treatment for at least 4 weeks, and age-matched healthy volunteers were enrolled in the study after providing written informed consent. 6 mm punch biopsies were taken from the abdomen under local anesthesia, embedded in optimal cutting temperature compound O.C.T.™ (Tissue-Tek®, Sakura Finetek, Zoeterwoude, Netherlands), and stored at −80°C until further processing. Non-lesional biopsies were taken 2 cm distant from the margin of a chronic psoriasis plaque. 7-μm cryosections were fixed in acetone and incubated with a mouse anti-Langerin or an anti-BDCA-2 antibody in PBS with 2% BSA overnight at 4°C. After incubation with 1% H_2_O_2_ for 10 min, antibody binding was visualized using conventional immunohistochemical staining (Dako REAL™ Detection Systems HRP/AEC, Dako AutostainerLink 48, Dako, Glostrup, Denmark). For LC and pDC quantification, immunohistological images were acquired using a Zeiss Observer.Z1 microscope (Carl Zeiss, Oberkochen, Germany) equipped with TissueFAXS® and 2 sites per sample were analyzed using HistoQuest® software (both Tissue Gnostics, Vienna, Austria).

### Scoring of the psoriatic phenotype

To monitor psoriasis severity of individual mice, a psoriasis severity scoring system modified from Singh *et al* (2010) was used rating the degree of erythema, swelling, and scaling of the skin separately for five dermatomes (ears, tail, paws, snout, back skin). We attributed a score of 0–4 to each of the dermatomes, defining a score of 0 as absence of pathological symptoms, 1 as isolated, sparse lesions or visible rubor, 2 as several lesions accompanied by low-grade swelling, 3 as moderate inflammation of most parts of the dermatome, and a score of 4 as intense swelling, redness and scaling of the complete dermatome and the absence of healthy skin. The phenotype score was attributed to each dermatome of each mouse in a blinded fashion and summarized as a cumulative score.

### Bone marrow chimeric mice

Host CD45.2 mice were exposed to whole body gamma irradiation, applying the lethal dose of 10 Gray. Subsequently, CD45.1 donor bone marrow cells were isolated, and T cells were depleted either via biotinylated antibodies against CD3 (Biolegend) and CD90 (Biolegend), followed by negative magnetic sorting with IMag™ streptavidin-coated magnetic beads (BD Biosciences), or using MACS CD3 microbeads (Miltenyi) according to the manufacturer's protocol. Of 3.5 × 10^6^ bone marrow cells (depleted of T cells) were injected into the tail vein of each host animal, and mice were maintained for 6 weeks on acidified water. Subsequently, chimerism was verified in peripheral blood collected from the tail via flow cytometric analysis of CD45.1 and CD45.2. Chimerism was routinely > 90%.

### Isolation of cells from epidermal, dermal, lymph node, and splenic suspensions

Mice were euthanized by cervical dislocation, and skin cells were isolated from ears and tails. Dorsal and ventral ear halves were separated, and tail skin was peeled from residual tissue. Skin sheets were then placed on 0.8% trypsin for 45 min (Fisher Scientific) at 37°C. Epidermis and dermis were separated, and epidermal pieces were incubated for 30 min at 37°C in PBS containing 8% FCS (PAA) and 100 μg/ml DNase I (Sigma-Aldrich). Dermal pieces were incubated in PBS with 1% FCS, 100 μg/ml DNase I, and 100 μg/ml Liberase TM (Roche) for 30 min at 37°C. Epidermal and dermal cell suspensions for flow cytometric analysis shown in Fig 2B were isolated as previously described (Tamoutounour *et al*, 2013). Auricular lymph nodes and spleen were isolated and incubated for 30 min at 37°C in PBS supplemented with 1% FCS, 100 μg/ml DNase I and 50 μg/ml Liberase TM. After red blood cell lysis, suspensions were filtered through a 70-μm cell strainer (BD Biosciences). Spleens were flushed with PBS containing 1% FCS, 100 μg/ml DNaseI, and 50 μg/ml Liberase TM and incubated in this enzyme mix for 30 min at 37°C.

### Flow cytometry

Single cell suspensions were stained with fluorescent antibodies for 30 min on ice after blocking Fc-receptors with anti-CD16/CD32 antibody. For intracellular IL-17 staining of DKO* skin, dermal and epidermal cell suspensions were pooled and stimulated for 4.5 h with 500 ng/ml PMA (Sigma) and 500 ng/ml ionomycin (Sigma) in the presence of GolgiPlug (BD Biosciences) for the last 4 h.

For a list of monoclonal antibodies used, see Supplementary Table S1. Gating for flow cytometric analysis in Fig 2B was performed as previously described (Tamoutounour *et al*, 2013). In brief, subsets were gated as:

CD11b^+^DCs (CD11b^+^CD64^−^CCR2^+^Ly-6C^−^MHC-II^+^ CD24^lo^)

MHC-II^lo^moDCs (CD11b^+^CD64^lo^CCR2^+^Ly-6C^hi^MHC-II^lo^ CD24^−^)

MHC-II^+^moDCs (CD11b^+^CD64^lo^CCR2^+^Ly-6C^lo^MHC-II^+^ CD24^−^)

MHC-II^+^ dermal macrophages (CD11b^+^CD64^hi^CCR2^lo^Ly-6C^lo^MHC-II^+^ CD24^−^)

Dead cells were excluded by fixable dead cell stainings (Fisher Scientific, ebioscience). For intracellular stainings, cells were fixed in 2% PFA (Roth) and subsequently permeabilized using PermWash buffer (BD Biosciences). For Ki67, IL-17, and FoxP3 stainings, a FoxP3 Fix/Perm buffer set (Biolegend) was used. Flow cytometry was performed on a LSRFortessa cell analyzer (BD Biosciences), and data were analyzed with FlowJo 7.6.4 software (Treestar). All flow cytometric gatings were performed on live cells following exclusion of doublets with FSC-A/FSC-H. Gates for activation markers and intracellular FoxP3, BrdU, Ki-67, and IL-17 stainings were set according to a corresponding isotype control. Numbers in flow cytometric plots and within graphs depicting quantifications of flow cytometric stainings indicate the percentage of a population of live single cells.

### *In vivo* BrdU labeling

For proliferation studies of LCs, mice received one intraperitoneal injection of 1.5 mg 5-Bromo-2′deoxyuridine (BrdU, Calbiochem) followed by 1 week of BrdU application via drinking water (0.8 mg/ml). BrdU content was analyzed by flow cytometry using a BrdU Flow Kit (BD Biosciences).

### Imiquimod treatment

Ears and/or shaved back skin of 8- to 12-week-old C57BL/6 mice were treated topically with Aldara, a 5% Imiquimod cream formulation every other day for 14 days, as previously described by our group (Drobits *et al*, 2012), resulting in a total of 7 imiquimod applications. Alternatively, for the data shown in Supplementary Figs S2K–M and S4D–I, Imi was applied daily on 6 consecutive days according to the treatment regimen described by van der Fits (van der Fits *et al*, 2009) and mice were analyzed on day 7.

### Skin thickness measurement

Ears were cut off at the base and split in half, and the lower ear half was embedded in paraffin. 4-μm sections were stained with hematoxylin and eosin (Sigma-Aldrich). Images were obtained with a Nikon eclipse 80i microscope; histomorphometric analysis was performed using the Lucia system. Epidermal and dermal thickness were measured on 10 random fields on 3–4 independent pictures per sample, magnification 4×, using Adobe Photoshop CS4 (Adobe).

### Immunofluorescence stainings

Tissues were embedded in O.C.T.™ (Sakura), and 5-μm cryosections were generated and fixed in acetone before processing. Epidermal ear sheets were generated by separating epidermis from dermis with 3.8% ammoniumthiocyanate (VWR) and fixed in 4% PFA (Roth). Samples were blocked for 30 min at room temperature in 1% bovine serum albumin (Sigma-Aldrich) in PBS containing 5% goat serum (PAA) and 0.1% Triton (Sigma-Aldrich) and were incubated with the indicated antibodies overnight at 4°C in the same buffer. Apoptosis of epidermal LCs was assessed by co-staining with antibodies against Langerin and active caspase-3 followed by a secondary staining with the DyLight 594 goat anti-rabbit IgG (Vector Laboratories).

### Total RNA isolation and RT–PCR analysis of murine cells and tissues

Total RNA from epidermal cells was isolated with TRIzol Reagent (Invitrogen). Complementary DNA (cDNA) synthesis was performed with SuperScript First-Strand Synthesis System (Invitrogen) according to the manufacturer's instructions. qRT–PCRs were carried out using SYBR Green Mix (Applied Biosystems), according to the manufacturer's instructions. For a list of primer sequences employed, see Supplementary Table S2. PCRs were performed on a 7500 Fast Real-Time PCR System (Applied Biosystems, California USA) under the following conditions: an initial incubation at 50°C for 20 s and 95°C for 10 min followed by 40 cycles of 95°C for 15 s, 54°C for 1 min. Relative quantification of RNA was calculated by ΔΔ*C*_t_ method. Omission of cDNA or reverse transcriptase enzyme was used as negative controls.

### Isolation of LCs and epidermal cells

LCs were collected as previously described (Holcmann *et al*, 2009).

### *In vivo* inhibition of IL-23R signaling

The monoclonal antibody to mouse IL-23R (21A4) was generated at Merck Research Laboratories (Palo Alto). To inhibit IL-23 signaling, mice with established disease were treated by either intraperitoneal injection with 300 μg anti-IL23R antibody or an isotype mouse IgG1 antibody (27F11) every other day. Mice were grouped randomly, and phenotype score was assessed weekly. Ears were analyzed by histology, and tail skin was used for flow cytometry.

### Microscopy

Confocal microscopic pictures were acquired on a Zeiss LSM700 and evaluated using the ZEN2010 software.

### Graphs and statistics

Experiments were performed at least two times, and data are represented as mean ± standard error of the mean (SEM). All graphs and statistical analyses were generated GraphPad Prism4 and Adobe illustrator software. Unpaired two-tailed student's *t*-test, Mann–Whitney *U*-test, and Wilcoxon signed-rank test were used to assess statistical significance (*P < 0.05, **P < 0.01, ***P < 0.001), as indicated in the figure legends.

**The paper explained**

**Problem**

Psoriasis is a frequent inflammatory skin disease of unknown etiology characterized by dramatic changes in the composition of the skin's inflammatory cells. Among these are neutrophils and T cells, and several types of dendritic cells (DCs), such as Langerhans cells (LCs) and plasmacytoid DCs (pDCs). Here, we investigated the function of these two types of DCs in the initiation and progression of psoriasis.

**Results**

We found a remarkable increase of pDCs in both the lesions of psoriatic patients and mouse models of psoriasis. In contrast, the resident epidermal LCs were dramatically decreased within lesional psoriatic plaques compared to healthy skin in both patients and mouse model. Using psoriatic mice, depletion of pDCs before the onset of psoriasis attenuated its severity in mouse models. However, during disease progression, psoriasis symptoms could not be ameliorated by pDC depletion. LC depletion experiments revealed that they were not involved in the initiation phase of psoriasis, but that aggravation occurred if LC were depleted during active disease.

**Impact**

Our results indicate that different DC subsets exert different functions during initiation and progression of psoriasis. Importantly, the resident epidermal LCs, which are gradually lost from the epidermis in the active disease phase, are responsible for keeping a suppressive skin environment via balancing the anti-inflammatory IL-10 and pro-inflammatory IL-23 axis. In future, this finding could be therapeutically explored. By supporting and strengthening the local LC network, the progression of psoriatic lesions might be prevented. This is especially important since the currently applied systemic treatments are associated with frequent side effects and are a burden for the health care system.
