# Supplementary material for: Specific roles for dendritic cell subsets during initiation and progression of psoriasis
Source: EMBO Mol Med. 2014 Sep 12;6(10):1312–27. doi: 10.15252/emmm.201404114 (PMC4287934; doi:10.15252/emmm.201404114)
Supplement: Supplementary file 1 [file emmm0006-1312-sd1.pdf]

TABLE OF CONTENTS

Supplementary information to

Specific roles for dendritic cell subsets during initiation and progression of psoriasis

Elisabeth Glitzner[1], Ana Korosec[1], Patrick M. Brunner[2], Barbara Drobits[1], Nicole Amberg[1], Helia B. Schonthaler[3], Tamara Kopp[2], Erwin F. Wagner[3], Georg Stingl[2], Martin Holcmann[1], and Maria Sibilia[1*]

Supplementary Fig. 1-6……………………………………………..…….p.1-6

Supplementary Table 1……………………………………….……….p.7

Supplementary Table 2……………………………………..…………….p. 8

Supplementary Figure legends………………………………......…….p.9-14

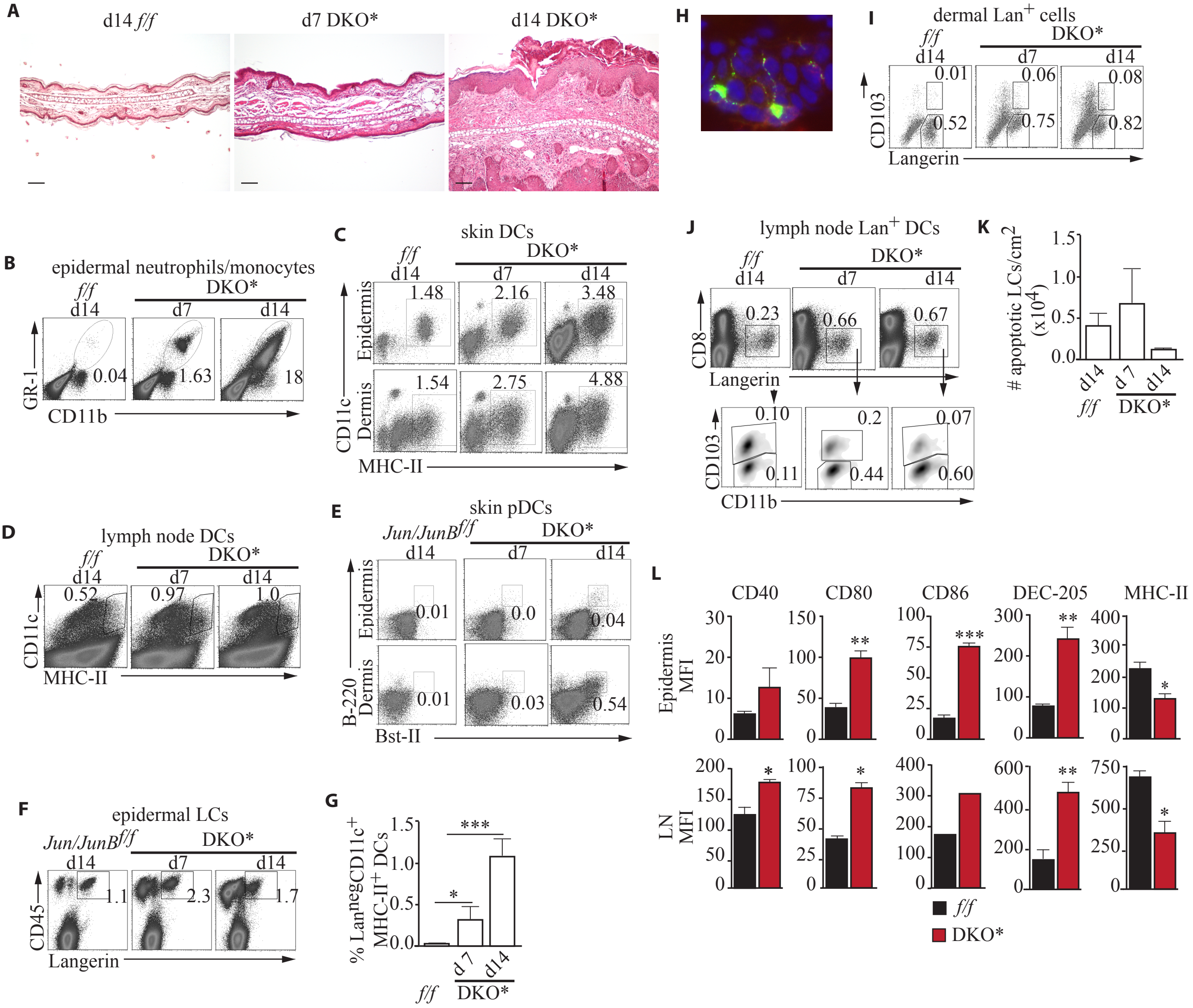

Supplementary Figure 1 Glitzner et al 2014

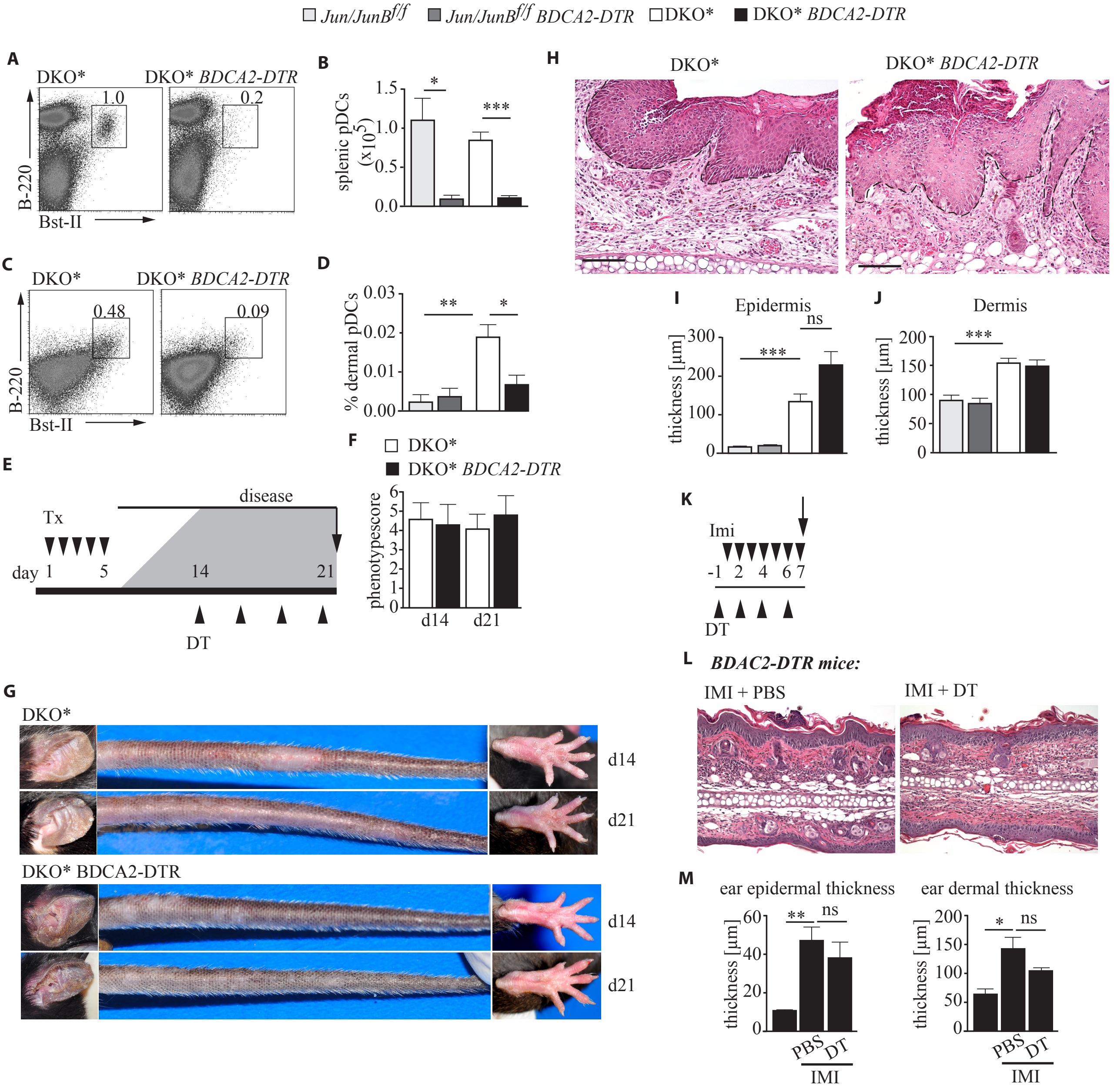

Supplementary Figure 2 Glitzner et al 2014

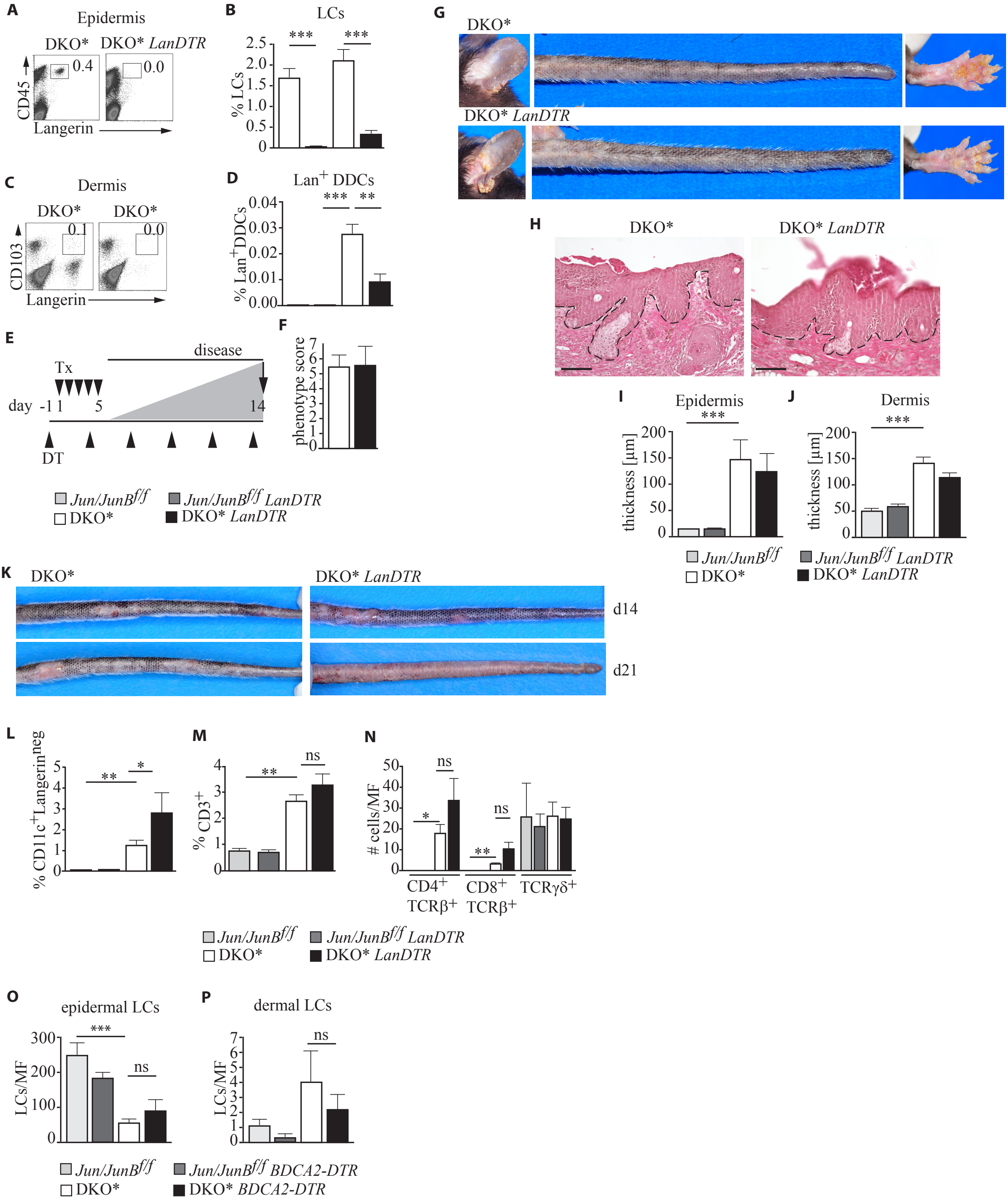

Supplementary Figure 3 Glitzner et al 2014

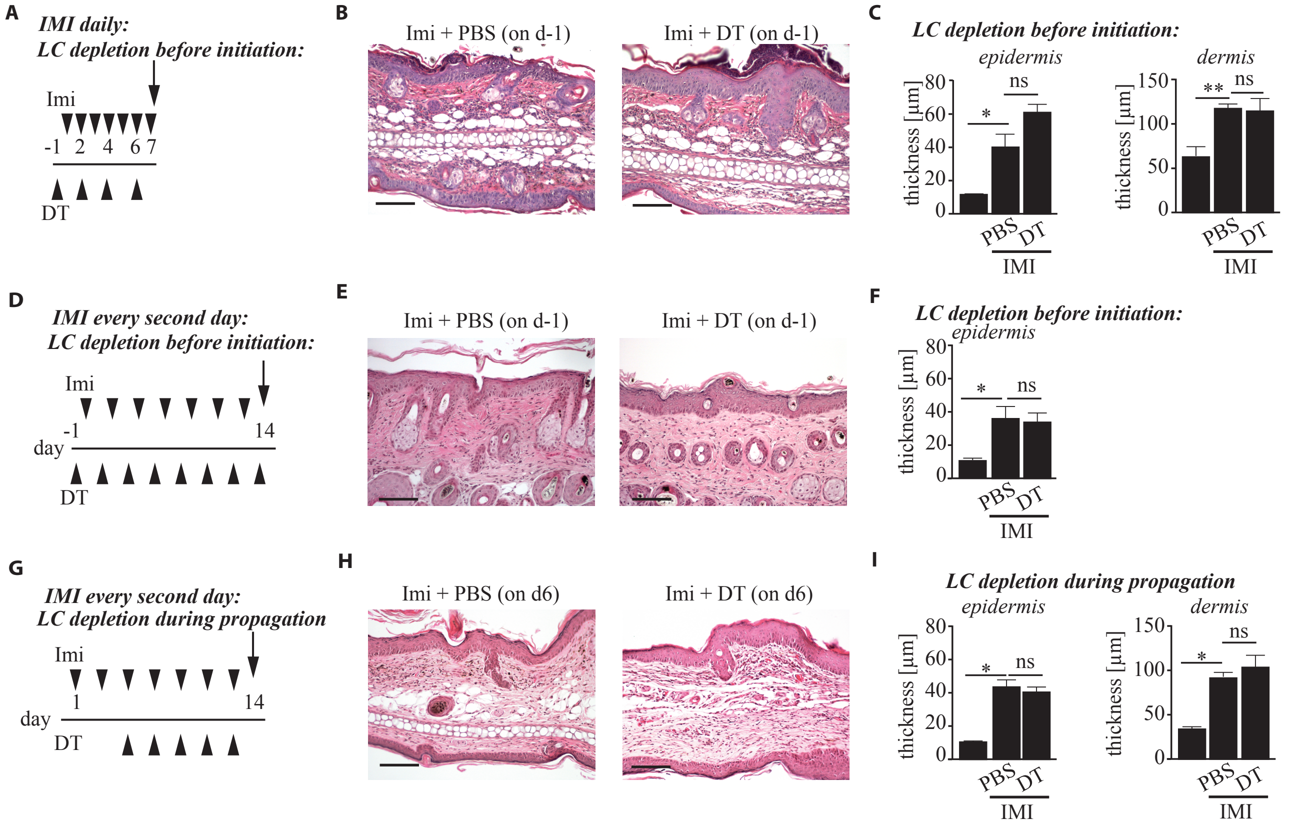

Supplementary Figure 4 Glitzner et al. 2014

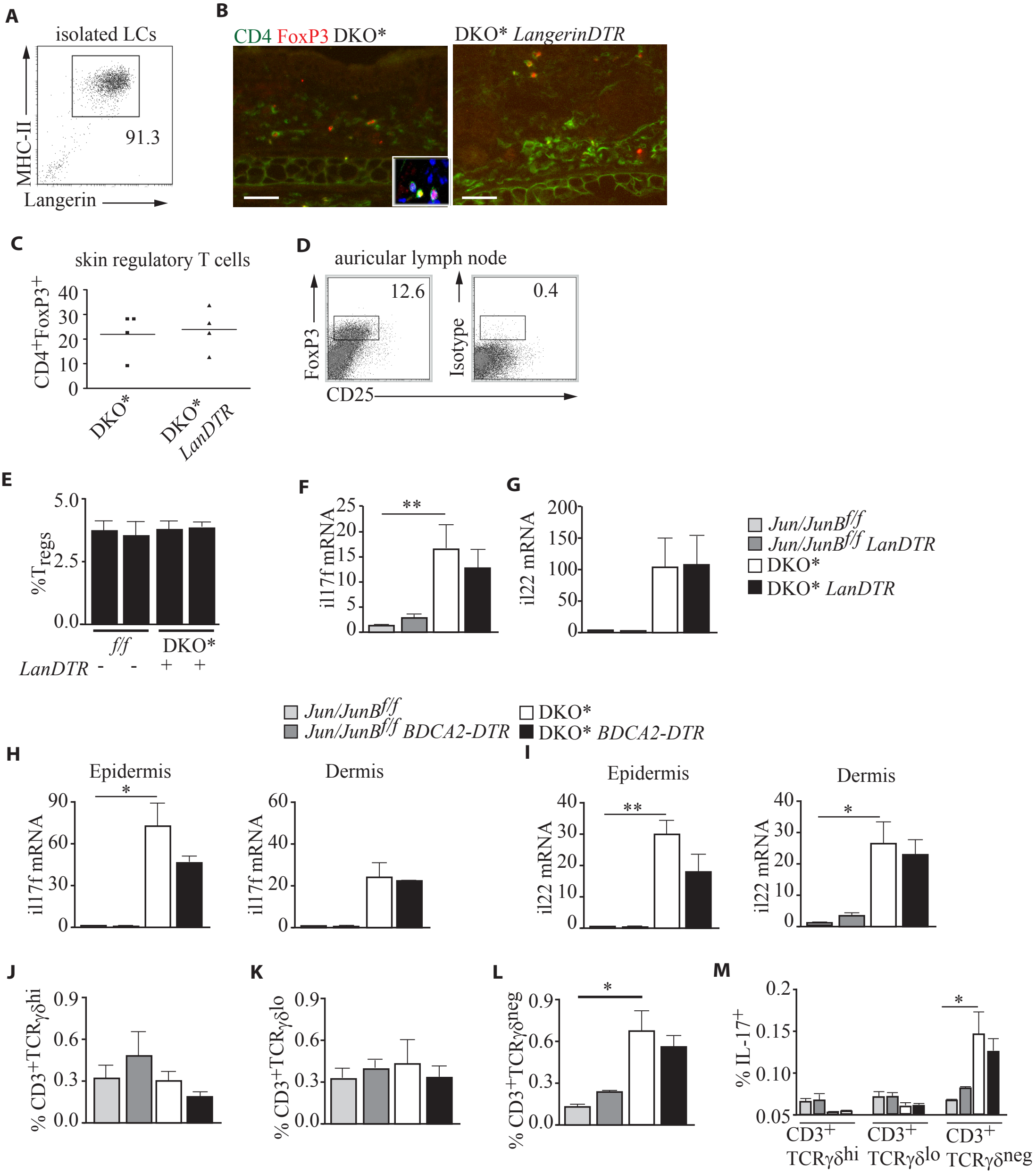

Supplementary Figure 5 Glitzner et al 2014

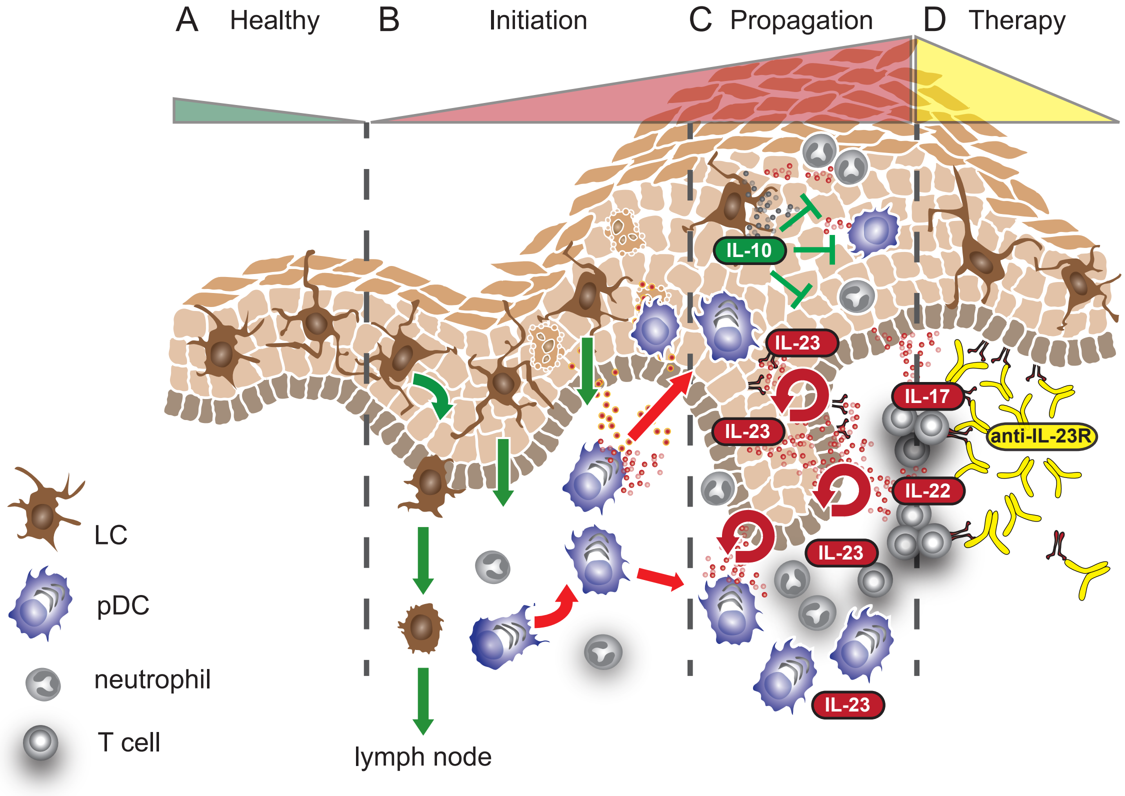

Supplementary Figure 6 Glitzner et al 2014

Antigen	Clone	Company
**Anti-mouse antibodies:**		
Bst-II	927	Biolegend
B-220	RA3-6B2	Biolegend
CCR2	475301	Millipore
CD3ε	145-2C11	Biolegend
CD4	GK1.5	Biolegend
CD8α	53-6.7	Biolegend
CD11b	M1/70	Biolegend
CD11c	N418	Biolegend
CD16/CD32	93	Biolegend
CD24	M1/69	Biolegend
CD40	3/23	Biolegend
CD45	30F11	Biolegend
CD45.1	A20	Biolegend/ebioscience
CD45.2	104	Biolegend
CD64	X54-5/7.1	Biolegend
CD80	16-10A1	Biolegend
CD86	GL-1	Biolegend
CD103	2E7	Biolegend
CD205	NLDC-145	Biolegend
GR-1	RB6-8C5	ebioscience
I-A/I-E	M5/114.15.2	Biolegend
IL-17a	TC11-18H10.1	Biolegend
Langerin	929F3.01	Dendritics
Ly-6C	HK1.4	Biolegend
Ki-67	B56	BD Bioscience
PD-L1	10F.9G2	Biolegend
TCRβ	H57-597	Biolegend
TCRγδ	UC7-13D5	Biolegend
mouse IgG1K	MOPC-21	BD Biosciences
rat IgG2bK	eB149/10H5	ebioscience
rat IgG2aK	eBR2a	ebioscience
Cleaved caspase-3	Accession No. #P42574	R&D Systems
**Anti-human antibodies:**		
BDCA-2	104C12.08	Dendritics
Langerin	DCGM4	Beckman Coulter

Supplementary Table 1: Antibodies used for flow cytometric stainings, immunofluorescence and immunohistochemistry

Transcript	Forward primer	Reverse primer
TATA-binding protein (TBP)	5'-GGGGAGCTGTGATGTGAAGT-3'	5'-CCAGGAAATAAT TCTGGCTCAT-3'
β-Actin	5'-CGGTTCCGATGCCCTGAGGCTCTT-3'	5'- CGTCACACTTCATGATGGAATTGA-3'
IL-10	5’-CAGAGCCACATGCTCCTAGA-3’	5‘-GTCCAGCTGGTCCTTTGTTT-3’
IL-17f	5'-TGCTACTGTTGATGTTGGGAC-3'	5'-AATGCCCTGGTTTTGGTTGAA-3'
IL-22	5'-AGCAGGTGCTCAACTTCACC-3'	5'-TGGATGTTCTGGTCGTCACC-3'
IL-23 p19	5'-CAGCTCTCTCGGAATCTCTGC-3'	5'-TGGCTGTTGTCCTTGAGTCC-3'
Mx1	5'-GAGCAAGTCTTCTTCAAGGATCA-3'	5'-GGGAGGTGAGCTCCTCAGT-3'

Supplementary Table 2: Primer sequences used for quantification of mRNA transcripts via quantitative real-time PCR.

Supplementary Fig. 1 - The dendritic cell network in DKO* mice. (A) Representative H&E staining of ear sections of indicated mice. Scale bars indicate 100 µm. Flow cytometric analysis showing (B) epidermal neutrophils/monocytes (CD11b^{+}GR-1$^{lo\text{-}hi}$ cells), **(**C) dermal and epidermal DCs (plots are gated on CD45^{+} cells), **(**D) migratory DCs (CD11c^{+}MHC-II^{hi}) in the auricular lymph node, **(**E) dermal and epidermal pDCs (CD45^{+}CD11c^{lo}B-220^{+}Bst-II^{+}CD11b^{+} cells) (plots gated on CD45^{+} cells) and **(**F) epidermal LCs (CD45^{+}Lan^{+}) of indicated mice. (G) Quantification of epidermal Lan^{neg} APCs (CD11c^{+}MHC-II^{+}Lan^{neg}) in auricular lymph nodes measured by flow cytometry (n=7-16). **(**H) Representative ear section of a day 14 DKO* mouse stained with Lan (green) and CD11c (red). Nuclear staining with HOECHST (blue). Flow cytometric analysis showing **(**I) dermal LCs and Lan^{+} DDCs (gated on CD45^{+}CD11c^{+} cells) and (J) LCs (CD8^{neg}Lan^{+}CD11b^{+}CD103^{neg} cells) in auricular lymph nodes of indicated mice. Lower plots are gated on Lan^{+}CD8^{-} cells. **(**K) Numbers of apoptotic LCs counted on at least 5 randomly chosen fields of images of ear sheets, (n=5-6). Magnification 10x. (L) Expression levels of activation markers on LCs isolated from epidermis (upper panel) or auricular LN (lower panel) (n=1-3). Numbers in flow cytometric plots indicate percentage of live single cells. Data represent mean +/- SEM. Data were analyzed using unpaired Student‘s t-test. (*p<0.05, **p<0.01, ***p<0.001). P values for this figure are available in Supplementary Table 3. Statistical source data for this figure can be found in the Source data section.

Supplementary Fig. 2 - pDCs are dispensable during the progression phase of psoriatic disease. (A-D) Efficiency of pDC depletion in DKO* mice treated according to the regimen depicted in Fig. 3A. (A) Flow cytometric analysis showing splenic pDCs (CD45^{+}CD11c^{+}B-220^{+}Bst-II^{+}CD11b^{neg} cells) and **(**B) quantification of splenic pDCs in indicated mice (n=5-8). **(**C) Flow cytometric analysis showing dermal pDCs (CD45^{+}CD11c^{+}B-220^{+}Bst-

II^{+}CD11b^{neg}cells) (plots gated on CD45^{+} cells) and (D) dermal pDC quantification in indicated mice (n=3-7). (E) Psoriatic disease was induced by five daily consecutive injections of Tx (▼), and on day 14, when psoriatic disease had developed, pDCs were depleted by injection of DT (▲) (applied every other day). Mice were analyzed on day 21. (F) Mean psoriatic phenotype score of the indicated mice was determined on day 14 and day 21 after disease induction (n=9-13). (G) Representative images of affected body parts of indicated mice on day 14 and day 21. (H) Representative H&E staining of ear sections of indicated mice on day 21. Scale bars indicate 100 µm. Dashed line indicates epidermal-dermal junction. (I) Histogram showing epidermal and (J) dermal thickness of skins of the indicated genotypes. 10 randomly chosen fields of 3-4 independent images per mouse were analyzed (n=4-8). Magnification 4x. (K-M) *BDCA2-DTR* mice were injected either with PBS or DT (▲) starting on d -1 every other day, and treated daily for 6 days with Imi (▼) on ears. Experiment was terminated on d 7 (K). (L) Representative H&E stained ear skin sections of indicated mice are shown. (M) Histogram showing epidermal and dermal thickness of ears of mice of the indicated genotype. (n=3). Data represent mean +/- SEM. 10 randomly chosen fields of 3-4 independent images per mouse were analyzed, magnification=4x. Data were analyzed using unpaired Student‘s t-test (*p<0.05, **p<0.01, ***p<0.001).Flow cytometric quantifications are depicted as percentage of live cells. *Jun/JunB$^{f/f}$* (light grey), *Jun/JunB$^{f/f}$ BDCA2-DTR* (dark grey), DKO* (white) and *DKO*BDCA2-DTR* (black). Data represent mean±SEM. Data for B and D were analyzed using unpaired Student’s T-test, and for I and J using Mann-Whitney U Test (F, I, J) (*p<0.05, **p<0.01, ***p<0.001). P values for this figure are available in Supplementary Table 3. Statistical source data for this figure can be found in the Source data section.

Supplementary Fig. 3 - Lan$^+$ APCs are dispensable for initiation of psoriatic disease, but counteract chronic psoriatic inflammation. (A-D) Efficiency of Lan$^+$ APC depletion in DKO* mice treated according to Fig. 4A. Mice were treated 14 days after psoriatic disease induction for 1 week with DT. Flow cytometric analysis (A) and (B) quantification of epidermal LCs (Lan$^+$CD45$^+$ cells) (n=9-12). (C) Flow cytometric analysis and (D) quantification of dermal Lan$^+$ DDCs (CD45$^+$CD103$^+$Lan$^+$ cells, plots gated on CD45$^+$ cells) (n=4-8). (E-P) Role of Lan$^+$ APCs during disease initiation. (E) Mice were injected with DT (▲) every third day starting 1 day prior to Tx (▼) and euthanized on day 14 after disease induction. (F) Mean psoriatic phenotype score of the indicated mice was determined on day 14 after disease induction (n=12-16). (G) Representative images of affected body parts of indicated mice. (H) H&E stained ear sections of indicted mice. Scale bars indicate 100 µm. Dashed line indicates epidermal-dermal junction. (I) Histograms showing epidermal and (J) dermal thickness of skins of the indicated genotype. 10 randomly chosen fields of 3-4 independent images per mouse were analyzed (n=8-14). Magnification 4x. (K-N) Experiments were performed as indicated in Fig. 4A. (K) Representative images of affected body parts of DKO* and DKO* *LanDTR* mice one week after the first DT injection. (L) Flow cytometric analysis showing epidermal Lan^{neg} CD11c$^+$ APCs (CD45$^+$CD11c$^+$Lan^{neg} cells) (n=11-18) and (M) T cells (CD45$^+$CD3$^+$) (n=8-12). (N) CD4$^+$ T cells (TCRβ$^+$CD4$^+$), CD8$^+$ T cells (TCRβ$^+$CD8$^+$) and γδ T cells (TCRγδ$^+$) counted on at least 5 randomly chosen fields of ear sections of indicated mice (n=3-9). *Jun/JunB$^{f/f}$* (light grey), *Jun/JunB$^{f/f}$ LanDTR* (dark grey), DKO* (white) and DKO* *LanDTR* (black) mice. (O,P) Experiments were performed as indicated in Fig. 3A. (O) epidermal and (P) dermal LCs (Langerin$^+$CD103^{neg} cells) were counted on at least 5 randomly chosen fields of ear sections of indicated mice (n=4-6). *Jun/JunB$^{f/f}$* (light grey), *Jun/JunB$^{f/f}$ BDCA2-DTR* (dark grey), DKO* (white) and DKO* *BDCA2-DTR* (black) mice. Flow cytometric quantifications are depicted as percentage of live

cells. Data represent mean +/- SEM. Data for B, D, N, O and P were analyzed using unpaired Student‘s t-test, for F, L and M using Wilcoxon signed-rank test and for I and J using Mann-Whitney U test (*p<0.05, **p<0.01, ***p<0.001). P values for this figure are available in Supplementary Table 3. Statistical source data for this figure can be found in the Source data section.

Supplementary Fig. 4 - LC or pDC depletion before or during Imiquimod (Imi) treatment does not influence skin inflammation.). (A) LanDTR mice were injected either with PBS or DT (▲) starting on d -1 every other day, and treated daily for 6 days with Imi (▼) on ears. Experiment was terminated on d 7. (B) Representative picture of H&E stained ear skin sections of indicated mice are shown. (C) Histogram showing epidermal and dermal thickness of mice of the indicated genotype (n=3). (D) *LanDTR* mice were injected either with PBS or DT (▲) starting on d -1 twice weekly and treated every other day for 14 days with Imi (▼) on the back skin. (E) H&E stained back skin sections are shown. (F) Histogram showing epidermal thickness of mice of the indicated genotype (n=4-5) (G) LanDTR mice were treated every other day for 14 d with Imi on ears. On d5, when inflammation was evident, DT or PBS was injected every third day. (H) H&E stained ear sections of indicated mice are shown. (I) Histograms showing epidermal and dermal thickness of mice of the indicated genotype (n=3-5) Data represent mean +/- SEM. 10 randomly chosen fields of 3-4 independent images per mouse were analyzed magnification=4x. Data were analyzed using unpaired Student‘s t-test (*p<0.05, **p<0.01, ***p<0.001). P values for this figure are available in Supplementary Table 3. Statistical source data for this figure can be found in the Source data section.

Supplementary Fig. 5 - LC or pDC depletion impact on immunological skin and lymph node priming. (A) Flow cytometric analysis showing LCs isolated from epidermal ear sheets of DKO* mice on day 8 after disease induction. **(**B) Representative pictures of ear sections of indicated mice stained with CD4 (green) and FoxP3 (red) (Inlay: additional staining with HOECHST). **(**C) $CD4^{+}FoxP3^{+}$ cells counted on at least 8 randomly chosen fields of ear sections (n=4). Magnification 10x. **(**D) Flow cytometric analysis showing auricular lymph node Tregs. Gated on $CD3^{+}CD4^{+}$ cells. **(**E) Number of Tregs ($CD3^{+}CD4^{+}CD25^{+}FoxP3^{+}$ cells) determined via flow cytometry in auricular lymph nodes of indicated mice (n=3-7). **(**F) Relative *IL17f* (n=7-8) and **(**G) *IL-22* mRNA (n=6-8) expression in epidermal cells of the indicated mice treated as depicted in Fig. 4A. **(**H) Relative epidermal (n=3-4) and dermal (n=3-7) *IL17f* and **(**I*)* epidermal (n=1-3) and dermal (n=3-7) *IL-22* mRNA expression in epidermal and dermal cells of the indicated mice treated as depicted in Fig. 3A. (J) Quantification of flow cytometric analysis showing $CD45^{+}CD3^{+}TCR\gamma\delta^{neg}$, (K) CD45+CD3+$TCR\gamma\delta^{neg}$, $CD45^{+}CD3^{+}TCR\gamma\delta^{hi}$ (L) $CD45^{+}CD3^{+}TCR\gamma\delta^{lo}$ cells and (M) IL-17 production by these subsets in the skin of *Jun/JunB$^{f/f}$*, *Jun/JunB$^{f/f}$BDCA2-DTR*, DKO* and DKO* *BDCA2-DTR* mice (n=6-7). Data were analyzed using unpaired Student‘s t-test. (*p<0.05, ***p<0.001). Flow cytometric quantifications are depicted as percentage of live cells. Scale bars indicate 100 µm. Data represent mean +/- SEM. P values for this figure are available in Supplementary Table 3. Statistical source data for this figure can be found in the Source data section.

Supplementary Fig. 6 - Role of pDCs and LCs in psoriasis initiation and progression. (A) In healthy skin, LCs populate the epidermis in high numbers, whereas pDCs are absent. **(**B) During the initiation phase of psoriasis, LCs start to emigrate from the epidermis to the local draining lymph nodes, whereas pDCs infiltrate the dermis and epidermis. pDCs support the

initiation of the inflammatory cascade, leading to enhanced production of IL-23 and subsequent development of all psoriatic hallmarks. **(**C) In the propagation phase, LC numbers decrease in the epidermis, while remaining LCs dampen inflammation by local production of IL-10, thereby suppressing IL-23 production. **(**D) Therapeutic inhibition of IL-23 signaling (anti-IL-23R) blocks the inflammatory cascade leading to regression of psoriatic lesions. Bone marrow-derived LCs repopulate the epidermis, ultimately resulting in complete reestablishment of skin homeostasis.

Supplementary Table 1 – Antibodies used for immunofluorescence / immunohistochemistry / flow cytometry

Supplementary Table 2 – Primer sequences employed for quantitative real-time RT PCRs
