# Supplementary material for: Specific roles for dendritic cell subsets during initiation and progression of psoriasis
Source: EMBO Mol Med. 2014 Sep 12;6(10):1312–27. doi: 10.15252/emmm.201404114 (PMC4287934; doi:10.15252/emmm.201404114)
Supplement: Supplementary file 4 [file emmm0006-1312-sd4.pdf]

Specific roles for dendritic cell subsets during initiation and progression of psoriasis

Elisabeth Glitzner, Ana Korosec, Patrick M. Brunner, Barbara Drobits, Nicole Amberg, Helia B Schonthaler, Tamara Kopp, Erwin F. Wagner, Georg Stingl, Martin Holcmann and Maria Sibilia

Corresponding author: Maria Sibilia, Medical University of Vienna

Review timeline:

Submission date:	27 March 2014
Editorial Decision:	07 May 2014
Additional correspondence (author):	07 May 2014
Additional correspondence (editor):	12 May 2014
Revision received:	21 July 2014
Editorial Decision:	04 August 2014
Revision received:	12 August 2014
Accepted:	15 August 2014

Transaction Report:

(Note: With the exception of the correction of typographical or spelling errors that could be a source of ambiguity, letters and reports are not edited. The original formatting of letters and referee reports may not be reflected in this compilation.)

Editor: Céline Carret

1st Editorial Decision 07 May 2014

Thank you for the submission of your manuscript to EMBO Molecular Medicine. I am sorry that it has taken so long to get back to you on your manuscript.

While reviewers 2 and 3 delivered their evaluations in a timely manner, we did not receive the other reviewer's input yet who did ask to have more time. As the evaluations from the first two reviewers are consistent however, and a further delay cannot be justified, I have decided to proceed based on these evaluations. If in the meanwhile we should receive the other review within the next week, I will forward it to you straight away to be considered in your revision.

You will see that while both reviewers are generally supportive of your work and underline its potentials in terms of interest and novelty, they also raise a number of specific concerns that need to be fully addressed in the next version of your study.

Both referees are concerned that important conclusions are not fully supported by the data (technical and statistical issues). They do however constructively provide suggestions to address the problems raised in a satisfactorily manner and thereby improve clarity and conclusiveness of the manuscript. As the comments are well detailed and self-explanatory, I will not get into too much more details.

Should you be able to address the raised concerns with additional experiments where appropriate, we would be willing to consider a revised manuscript.

Please note that it is EMBO Molecular Medicine policy to allow a single round of revision in order to avoid the delayed publication of research findings. Consequently, acceptance or rejection of the

manuscript will depend on the completeness of your responses included in the next version of the manuscript.

EMBO Molecular Medicine has a "scooping protection" policy, whereby similar findings that are published by others during review or revision are not a criterion for rejection. Should you decide to submit a revised version, I do ask that you get in touch after three months if you have not completed it, to update us on the status.

Please also contact us as soon as possible if similar work is published elsewhere. If other work is published we may not be able to extend the revision period beyond three months.

I look forward to seeing a revised form of your manuscript as soon as possible.

***** Reviewer's comments *****

Referee #2 (Remarks):

In this manuscript, Glitzner and colleagues propose that plasmacytoid DCs (pDCs) contribute to disease during initiation of psoriasis by making IL-23, while Langerhans cells (LCs) dampen the inflammation during the chronic state of disease via production of IL-10. To support their hypothesis, the authors use two different mouse models of psoriasis, including mice with keratin 5-specific deletion of Jun and JunB (DKO mice) as well as the imiquimod model. The manuscript is well written and the model proposed by the authors is an attractive one. While some of the presented data (notably the BM chimeras) support their model, some elements of the hypothesis and central message of this manuscript is not supported by the data. Moreover, this reviewer expresses certain methodological concerns.

Major points:

1) Although their hypothesis and central message rests on it, the authors do not show IL-23 production by pDCs and IL-10 production by LCs in their model(s). The authors could address this issue by performing an intracellular cytokine staining followed by flow cytometry of DC subsets or by qPCR of purified DC subsets. An alternative approach could be the use of multiple staining immunohistochemistry or fluorescence microscopy and quantification.

2) The distinction of LCs vs. langerin+ dermal DCs (Lan+ DDCs) is not clear and leads to confusion. In Fig. 1, the authors suggest that increased numbers of LCs were present in lesional dermis, yet it is unclear for this reviewer how the authors conclude these are LCs, and not Lan+ DDCs. Given the central role of LCs in this study, the authors should provide additional evidence that these cells are indeed dermal LCs instead of Lan+ DDCs. The latter are known to exert anti-inflammatory properties via the production of IL-10. Likewise, it is confusing how the authors define the Lan+ DC subsets in their mouse model(s), with regard to the distinction between LCs and Lan+ DDCs as well as the expression of CD103 by these cells. In Fig. 2J, the authors define Lan+ DDCs as CD45+ Lan+ CD103+, whereas in Fig. 2I Lan+ DDCs are defined as Lan+ MHC-II+ CD11c+ CD103- CD8- cells, and are, based on these markers, identical to LCs (auricular LN-homing LCs were defined as Lan+ CD103- CD8- MHC-II+ CD11c+ cells in Fig. 2I).

3) The authors use a different protocol for inducing psoriasis inflammation using imiquimod (7 application every second day for two weeks) than the one they refer to and commonly used by others (6-7 daily applications for one week). The authors need to explain why they used this modification as this might alter the nature of the inflammatory infiltrate observed in their model. Moreover, the authors should provide quantitative data on ear or epidermal thickness upon imiquimod application in their different experimental groups, rather than simply showing a histological section. Such quantitative data would be more convincing, as for example in Suppl. Fig. 4D, the dermis appears to be thicker in DT-treated animals.

4) In Suppl. Fig. 3L, M, the authors describe that intraepidermal T cells were increased in Lan+ DC-depleted DKO mice. Previous reports have shown that CD8+ T cells and/or gamma-delta T cells might drive inflammation in psoriasis. The authors must have specified these intraepidermal T cells and should show whether they are CD4+, CD8+ or gamma-delta T cells.

Minor points:

a) On p. 9, it should presumably read Supplementary Fig. 2F, G.

b) The terms Lan+ DCs and Lan+ DDCs are confusing. The authors might consider using Lan+ APCs instead of Lan+ DCs.

c) The color legend to Fig. 5H seems incomplete and should be corrected.

d) In Suppl. Fig. 4L, M, the authors claim that the depletion of pDCs leads to reduced levels of IL-17 and IL-22 mRNA. However, the values shown do not seem to be statistically different. In addition, the differences in Suppl. Fig. 4J, K are comparable to those observed in Suppl. Fig. 4L, M, and yet the authors reach the opposite conclusion. The description of these panels should be revised to provide a more accurate representation of the actual data.

e) Some of the relevant literature on DC subsets in the used models has been omitted.

Referee #3 (Comments on Novelty/Model System):

1: Technical quality (type of experiments and analysis) is high. So is the statistical analysis. As indicated in the text, sometimes a trend but insignificant result is dealt with as it would be significant.
2: The novelty of the results for the particular model of psoriasis is high. the role of pDCs could have been expected based on previously published results in other models. the LC story is nice and novel, while the role of IL-23 is as expected from many other models.
3:

Referee #3 (Remarks):

In the present article, Glitzner and colleagues address the roles of pDCs and Langerhans cells in psoriasis resulting from tamoxifen-induced depletion of Jun/JunB genes in kerationcytes (DKO mice). Using the DTR/DT based cell depletion technology, they found that pDCs are required for initiation but not maintenance of disease, while LC prevent exacerbation of disease in the chronic phase without affecting induction of disease. They show data proposing that pDCs and LCs act by cross-regulation of IL-23. Notably, LCs seem not to regulate imiquimod-induced psoriasiform disease.

While an important role of pDCs in development of psoriasis has been established previously using a human - mouse xenograft psoriasis model, the authors not only confirm this result using another psoriasis mouse model but also extend the previous study by showing that pDCs are not required for maintenance of disease once fully established. Likewise, tolerogenic roles of LCs in skin inflammatory diseases have been established previously (as referenced properly by the authors of this ms), this study is the first to demonstrate novel a regulatory role of LCs in the chronic phase of psoriasis. Taken together the main results are interesting and convincing. Unfortunately, when it comes to the details some results are over-interpreted although the results seem not to be statistically significant.

Points to address.

1) In the experiments resulting to the conclusion that depletion of pDCs inhibits psoriasis (i.e. Fig 3), pDCs were depleted prior Tx-induced gene deletion and disease induction. The death of pDCs certainly changes the tissue microenviroment (e.g. phagocytosis of dead cells by DCs and macrophages, complement activation, lipid composition), which may secondary efficient induction of psoriasis. To exclude this possibility, I would suggest to first deplete Jun (2-3 consecutive Tx injection will certainly do it) followed by DT depletion of pDCs

2) Does pDC depletion affect numbers and localization of LCs?

3) Does pDC depletion affect gamma-delta T cell numbers and IL-17 production?

4) What is the role of pDCs in the imiquimoid psoriasis model ?

5) The statement that "The number of intraepidermal Langerin-negative infiltrating DCs as well as T cells were slightly increased in Lan+ DC-depleted compared to Lan+ DC-sufficient DKO* mice" should be omitted since there is no statistical significance. There is no such thing as slightly statistically significanct. (And of course, even a statistical significant difference doesn`t equate to biological importance).

6) The authors make a fuss about LC depletion resulting in massive IL-23 increase, as emphasized in the abstract). Yet, looking at Fig 7C, the increase in IL-23 comparing LC depleted and non-depleted DKO appears not to be significant !

7) Likewise anti-IL-23R treatment of DKO mice in the chronic phase of disease (d14-18) seems not to result in a significant reduction in disease score as compared to untreated DKO mice(Fig 7F). In their interpretation the authors compared a-IL-23R treated DKO at day 28 with untreated and DKO mice at day 14, which is insufficient. How often has this experiment been performed with similar results ?

8) The explanation for the differences in skin-resident and bone-marrow derived LCs in chronic (absence of Jun/JunB) vs non-chronic (imiquimoid treated) as determined in the bone marrow chimeras appears to me not really satisfactory. Mice were analyzed at day 9 (DKO) and day 8 (imiquimod-treated), which represents more or less the peak of disease when imiquiomod treatment is continued until then. There are probably differences in molecular requirements for development of psoriasis in the imiquimod vs Jun/JunB-deficiency model rather than simply one being chronic and the other not. I would suggest to extend imiquimod-treatment for another 7 days until day 14, in this case it could be considered as a chronic model and LCs play a role.

9) Figure 2 shows that DKO mice develop psoriasis peaking at d14. The authors state that this is associated with increased numbers of DCs in the lesions. However, shown are frequencies and not total numbers.

10) Gates set for characterization and quantitation for dermal DCs in
sup. Fig 1C are questionable. The gated population may consist of (at least) two populations including CD11c-negative (or low) and CD11c-intermedaite cell (both MHC class II positive). Cells should be stained with CD64 and F4/80 to distinguish tissue macrophages, DCs and inflammatory CD11c+ monocyes.

11) Frequency of CD11+ cells in the epidermis and dermis go up from d7 to d14. Yet LCs (Langerin+ cells in the epidermis) go down from d7 to d 14. Given that the large majority of CD11c+ cells in the epidermis are LCs, this observation does not make much sense.

12) Fig 2A, C,H, J show percentages, but it is not clear of what. Percentages of live CD45+ showed be shown. Given the low frequency of cells, especially pDCs and LCs, an experiment showing orginal FACS data with gating strategy, i.e. FSC vs SSC -> single cells > live cells -> CD45. And the autofluoresence should be gated out

While an important role of pDCs in development of psoriasis has been established previously using a human - mouse, the authors not only confirm this result using another psoriasis mouse model but also extend the previous study by showing that pDCs are not required for maintenance of disease once fully established. Likewise, tolerogenic roles of LCs in skin inflammatory diseases have been established previously (as referenced properly by the authors of this ms), this study is the first to demonstrate novel a regulatory role of LCs in the chronic phase of psoriasis. Taken together the main results are interesting and convincing. Unfortunately, when it comes to the details some results are over-interpreted although the results seem not to be statistically significant.

Additional correspondence (author) 07 May 2014

Many thanks for sending us the reviews of our manuscript. We are very happy that both the reviewers find our study interesting and novel.

We have carefully gone through their comments and we will be able to address them all and hopefully send you a revised version of the manuscript within the next few weeks.
In our study we are using complex genetic mouse models which we have kept breeding, so that if necessary we would have enough mice to perform the experiments requested by the reviewers. However, the number of available mice is not unlimited and therefore it would be great if we could get the comments of the third reviewer very soon so that we can plan all our in vivo experiments accordingly.
We will therefore wait until next Monday before starting with our mouse experiments.

There are also two questions related to reviewer 3 that we would like to get your opinion on:

1) We don't understand his/her first comment. There is a word missing, which precludes us from fully understanding what he/she really is concerned about. We can obviously perform the experiment he/she requests, but we do not understand the reasoning for requesting it. Maybe some word got lost during the "cut and paste" process?

2) In point 8, this reviewer request to generate again bone marrow chimaeras to analyse LC contribution to Imiquimod-treated skin at day 14 after treatment. We have shown these results for day 8 after Imiquimod treatment. We have not analysed them further because at day 14 there is spontaneous regression of Imiquimod-induced skin inflammation and therefore we do not think that the result would change. Therefore, we would like to avoid doing this experiment which would take around 12-14 weeks without adding significantly relevant information except that it would greatly delay the publication of our paper.

Additional correspondence (editor) 12 May 2014

Thank you very much for your email.
1st, I'd like to let you know that you can go ahead with the revision based on the 2 referees' reports you already have. While the third referee was specifically contacted and asked for putative additional comments, this referee agreed with the other 2 reports and would be happy with the suggested revision.

2nd, I have run your questions by referee 1 and please find the answers below:

"Regarding point 1: I am sorry for the confusion with the two typos. But the authors interpreted it correctly:

> which may secondarily affect induction of psoriasis"
> and if this is the case, the suggested experiment would answer this question

Regarding point 2: The authors suggestion (i.e. acute vs chronic model) for the differences observed in the dynamics of migrating and resident LCs in the two psoriasis models is rather unlikely given that the analysis was done more or less at the same time (days 8 and 9). Imiquimod treatment is usually performed until days 7-8 and mice analyzed one day later. (I'm actually not aware of a report that extended imq treatment beyond day 8). But I guess that pathology can be maintained until day 14 by continued treatment, which can then be considered as chronic (as the peak of disease is maintained for more than a week) and allow comparison to the chronic Jun-deficiency psoriasis model also at day 14.

This experiment is not absolutely essential for the main conclusion of the manuscript, but in its absence the authors have to tone down their explanation about the observed differences in LC dynamics and consider the possibility that the molecular pathways for the disease in the two models are not alike. "

I let you decide whether performing the experiment is necessary or not, but as suggested by the referee, if you don't, please rephrase your conclusions.

I hope this helps.

1st Revision - authors' response 21 July 2014

Reviewer #2:

We are very happy that this reviewer found the model proposed by our study attractive and the paper well written. We are thankful for the constructive criticism, which we have addressed as follows:

1) Although their hypothesis and central message rests on it, the authors do not show IL-23 production by pDCs and IL-10 production by LCs in their model(s). The authors could address this issue by performing an intracellular cytokine staining followed by flow cytometry of DC subsets or by qPCR of purified DC subsets. An alternative approach could be the use of multiple staining immunohistochemistry or fluorescence microscopy and quantification.

We had indeed measured IL-10 expression in LCs isolated from inflamed skin by qRT-PCR and had shown these results in Fig. 7A, in the manuscript on page (p.) 15. The purity of the isolated LCs was shown in Supplementary Fig. 4E, which has now become Supplementary Fig. 5A, since we added new results. We are sorry that the reviewer missed these results in the previous version of the paper. We also apologize for the misleading formulation of the IL-23 results. We observed that IL-23 is significantly increased in psoriatic epidermis of DKO* mice, but never implied that IL-23 is produced by pDCs (Fig. 7E). Since the IL-23 results were also questioned by Reviewer #3, we repeated all these experiments again with a new set of mice and can now demonstrate a significant reduction of IL-23 in the dermis of DKO* mice (and only slight, non-significant reduction in the epidermis) upon pDC depletion, which is depicted in Fig. 7F and described on p. 16 in the manuscript. Moreover, IL-23 is significantly increased in DKO* depleted of LCs, which show disease exacerbation. We are sorry if we did not make this clear in the original manuscript.

Since we found that in the epidermis, pDCs are not present at d0 (former Fig. 2C, now Fig. 2D), but they increase at day 14 when they are no longer relevant for psoriatic disease, we do not think that they play a major role in epidermal IL-23 production. Therefore, while we do not exclude that pDCs might produce IL-23 in psoriatic skin (and this might contribute to IL-23 reduction in their absence), we do not claim that IL-23 production is restricted to them. We have tried to detect IL-23, both at the transcriptional and protein level in pDCs, but were unable to reveal it. A previous study has located IL-23 production in psoriatic patient skin mainly to $CD14^{+}$ $CD11c^{+}$ $CD83^{neg}$ monocytes and $CD14^{neg}$ $CD11c^{+}$ $CD83^{+}$ DCs in the dermis (Lee et al, 2004). In mice, IL-23 production seems to be a cardinal feature of the subset of $CD11b^{+}$ DCs in the dermis (Wohn et al, 2013) as well as in other organs as the lung (Moreira et al, 2011) and the small intestine (Uematsu et al, 2008) (Schlitzer et al, 2013). We have restructured and reformulated the chapter describing these results on p. 15 and 16 and hope that it is now clearer.

2) The distinction of LCs vs. langerin+ dermal DCs (Lan+ DDCs) is not clear and leads to confusion. In Fig. 1, the authors suggest that increased numbers of LCs were present in lesional dermis, yet it is unclear for this reviewer how the authors conclude these are LCs, and not Lan+ DDCs. Given the central role of LCs in this study, the authors should provide additional evidence that these cells are indeed dermal LCs instead of Lan+ DDCs. The latter are known to exert anti-inflammatory properties via the production of IL-10.

Lan$^+$CD103$^+$ DCs are exclusively found in mice, but not in humans. While in humans, a subset of APCs is present in the dermis that exhibits functional overlap with mouse Lan$^+$ dermal DCs, they lack Langerin expression, and are instead identified by CD141 expression (Haniffa et al, 2012). Therefore, it is valid to state that Langerin-expressing DC subsets in the human dermis are LCs. We apologize if this point was not made clear and have now added a sentence in the introduction on p. 4 clarifying this issue.

Likewise, it is confusing how the authors define the Lan+ DC subsets in their mouse model(s), with regard to the distinction between LCs and Lan+DDCs as well as the expression of CD103 by these cells. In Fig. 2J, the authors define Lan+ DDCs as CD45+ Lan+ CD103+, whereas in Fig. 2I Lan+ DDCs are defined as Lan+ MHC-II+ CD11c+ CD103- CD8- cells, and are, based on these markers, identical to LCs (auricular LN-homing LCs were defined as Lan+ CD103- CD8- MHC-II+ CD11c+ cells in Fig. 2I).

We apologize for the confusion and would like to provide the following explanation: We generally defined Lan$^+$CD103$^+$ cells in the dermis as Langerin$^+$ dermal DCs (Lan$^+$DDCs), while Lan$^+$CD103^{neg} cells were defined as LCs, based on previous reports (Henri et al, 2010). In the lymph node, we stained for CD11b, as well as for CD8α as an exclusion marker in order to define the population of migratory Lan$^+$ dermal DCs (Langerin$^+$CD8^{neg} CD11b^{lo}CD103$^+$) and LCs (Langerin$^+$CD8^{neg}CD11b$^+$CD103^{neg}) (Henri et al, 2010) (see revised Supplementary Fig. 1J for gating). Therefore, we have changed the legend to Fig. 2J (formerly Fig. 2I) in our revised Figures, and it now reads "Lan$^+$ CD8^{neg} CD11b$^+$ CD103$^{\mathbf{neg}}$ cells" (can be found on p.36 in the manuscript).

3) The authors use a different protocol for inducing psoriasis inflammation using imiquimod (7 application every second day for two weeks) than the one they refer to and commonly used by others (6-7 daily applications for one week). The authors need to explain why they used this modification as this might alter the nature of the inflammatory infiltrate observed in their model. Moreover, the authors should provide quantitative data on ear or epidermal thickness upon imiquimod application in their different experimental groups, rather than simply showing a histological section. Such quantitative data would be more convincing, as for example in Suppl. Fig. 4D, the dermis appears to be thicker in DT-treated animals.

We have longstanding experience with topical imiquimod treatment of mice and have published two studies in this field, (Drobits et al, 2012), (Palamara et al, 2004) where we have established a protocol of imiquimod treatment every other day (also for animal welfare reasons). The effects obtained with our protocol and the daily treatment protocol are basically the same. We apologize for not having specified this. In order to convince this reviewer, we have also repeated the experiment according to the referee's suggestions (daily treatment) and show these results in Supplementary Fig. 4A-C. The reviewer can appreciate that the result is the same as shown in Supplementary Fig. 4D-F: LC depletion does not affect disease severity in the Imiquimod model. A similar result has recently also been reported by another group (Wohn et al, 2013).

As suggested by the reviewer, we have quantified the data of our previous experiments conducted with imiquimod treatment every other day (formerly Supplementary Fig. 4A-D) and show these results in Supplementary Fig. 4F and I. The description of these results has been added to the manuscript on p. 10-12 (results) and the imiquimod treatment modalities are now further specified in the Materials and Methods section on p. 25. We have also followed the referee's advice that the pictures previously chosen for Supplementary Fig. 4H (formerly Supplementary Fig. 4D) did not depict the actual situation very well. We have now replaced them with two more representative ones.

4) In Suppl. Fig. 3L, M, the authors describe that intraepidermal T cells were increased in Lan+ DC-depleted DKO mice. Previous reports have shown that CD8+ T cells and/or gamma-delta T cells might drive inflammation in psoriasis. The authors must have specified these intraepidermal T cells and should show whether they are CD4+, CD8+ or gamma-delta T cells.

We have addressed this point by performing immunofluorescence co-stainings on cryosections of ears, quantifying the number of $CD4^{+}TCR\beta^{+}$ or $CD8^{+}TCR\beta^{+}$, as well as $TCR\gamma\delta^{+}$ cells present in the epidermis of *jun/junB$^{f/f}$*, *jun/junB$^{f/f}$ LanDTR*, DKO* and DKO* *LanDTR* mice. We found that while γδ T cell numbers were not altered in DKO* mice, there was infiltration of mostly $CD4^{+}$, but also $CD8^{+}$ $TCR\beta^{+}$ T cells. While there is a clear trend for both these T cell specimens to be increased upon LC depletion, there is no statistical difference between LC-sufficient and LC-depleted DKO* mice. We show these data in Supplementary Fig. 3N, and have described them in the results on p. 12. We have furthermore included the antibodies employed in Supplementary Table 1.

Minor points:

a) On p. 9, it should presumably read Supplementary Fig. 2F, G.

We thank the referee for this comment and have corrected the error in the revised manuscript.

b) The terms Lan+ DCs and Lan+ DDCs are confusing. The authors might consider using Lan+ APCs instead of Lan+ DCs.

We agree that this terminology might be confusing and have now exchanged "Lan^{+}DCs" with Lan^{+}APCs throughout the manuscript.

c) The color legend to Fig. 5H seems incomplete and should be corrected.

We are grateful for pointing out this error and have corrected it.

d) In Suppl. Fig. 4L, M, the authors claim that the depletion of pDCs leads to reduced levels of IL-17 and IL-22 mRNA. However, the values shown do not seem to be statistically different. In addition, the differences in Suppl. Fig. 4J, K are comparable to those observed in Suppl. Fig. 4L, M, and yet the authors reach the opposite conclusion. The description of these panels should be revised to provide a more accurate representation of the actual data.

As already specified under point 1, all the results in this section were not well described and misleading. While we found that IL-23 levels are indeed significantly reduced in the dermis of DKO* mice upon pDC depletion, we neither found significant changes for epidermal or dermal IL-22 nor for IL-17f levels (previously Fig. 4 L and M, now found in Supplementary Fig. 5H and I). Accordingly, we quantified IL-17 producing T cells in DKO* mice depleted of pDCs, and found that while several IL-17 producing T cells (mostly of the $TCR\gamma\delta^{neg}$ subset) are present in the skin of DKO* mice, they are not significantly altered upon pDC depletion (Supplementary Fig. 5M). While it might sound counterintuitive that the IL-17/IL-22 axis is not changed, while IL-23 levels are reduced upon pDC depletion, also other groups have reported pathological effects of IL-23 that were independent of IL-17 in mouse models of asthma and colitis, which might be the case also here. We have reformulated the whole paragraph in the manuscript on p.16 and added a sentence in the discussion on p.19/20 and hope that is better now.

e) Some of the relevant literature on DC subsets in the used models has been omitted.

We had a lot of literature citations, which we had to cut back and therefore cited some reviews. We have followed the reviewer's advice and added back the original citations of some key papers describing DC subsets.

Reviewer #3:

This reviewer finds our results novel and the technical quality of our data high. We are thankful for his/her comments, which we have addressed as follows:

1) In the experiments resulting to the conclusion that depletion of pDCs inhibits psoriasis (i.e. Fig 3), pDCs were depleted prior Tx-induced gene deletion and disease induction. The death of pDCs certainly changes the tissue microenviroment (e.g. phagocytosis of dead cells by DCs and macrophages, complement activation, lipid composition), which may secondarily affect induction of psoriasis. To exclude this possibility, I would suggest to first deplete Jun (2-3 consecutive Tx injection will certainly do it) followed by DT depletion of pDCs.

We have now performed an experiment according to this referee's suggestions and found that pDC depletion, induced in an early stage of disease initiation (on day 2 after the first injection of Tx) again diminished the intensity of the psoriasis-like skin disease of DKO* mice. We have included a figure depicting these results for the editor and the referees (Attachment 1). The timeline is depicted in Panel A. Morphological disease symptoms as redness and inflammation were clearly reduced in DKO* *BDCA2-DTR* mice compared to DKO* mice (Panel B-D), and a significant reduction in ear epidermal thickness (Panel E) was seen, while dermal thickness was unchanged by pDC depletion (Panel F), recapitulating the findings that we reported in the first version of the manuscript in the context of pDC depletion before disease induction. We hope that this reviewer is now convinced that timing of *Jun/JunB* deletion does not change our conclusion. In addition, we have shown that depletion of LCs does not affect disease development in two models of psoriasis, and LCs are present at much higher numbers in the skin than pDCs, presumably leading to a much higher number of local apoptotic cells after DT treatment. We therefore think that it is unlikely that death of pDCs could affect disease development.

2) Does pDC depletion affect numbers and localization of LCs?

We have addressed this question by quantifying the number of LCs (defined as Langerin$^+$CD103$^-$ cells) present in the epidermis and the dermis on cryosections of ears of pDC-sufficient as well as pDC-depleted *jun/junB$^{f/f}$* and DKO* mice from 2 experiments and show these important results in Supplementary Fig. 3O and P. We confirm that the number of epidermal LCs is significantly reduced in DKO* mice at day 14, as it was already shown in Fig. 2G, and H of our original manuscript (which can now be found in Fig. 2H and I of our revised manuscript). However, we could not detect any significant difference in the number of LCs when pDCs were depleted in either control or DKO* mice, thereby excluding that pDC depletion influences the number of LCs at an early stage to contribute to disease initiation. We have described these data on p.12 of the results section and mentioned them on p.18 of the discussion.

3) Does pDC depletion affect gamma-delta T cell numbers and IL-17 production?

We have performed this experiment and are showing these results in Supplementary Fig. 5K-N. Furthermore, we have included the gating strategy for this experiment for the reviewers, in Attachment 2. Psoriatic DKO* mice have increased levels of TCR $\gamma\delta^{neg}$ T cells, which presumably represent TCR$\alpha\beta^+$ T cells, but γδ T cell numbers are unchanged when compared to controls. pDC depletion does not further affect both TCR $\gamma\delta^{neg}$ as well as γδ T cell numbers, which is similar to what is seen after LC depletion in the epidermis (see response to point 4, Reviewer # 2). IL-17 is increased in DKO* mice but is not significantly changed after pDC depletion. Interestingly, IL-17 does not seem to be produced by γδ T cells, but rather by γδ negative T cells in this model. We have mentioned these results on p16.

4) What is the role of pDCs in the imiquimoid psoriasis model?

A study addressing the influence of imiquimod-induced inflammation was recently published, which employed both constitutive pDC depletion (E2-$2^{-/-}$ mice) as well as conditional pDC depletion (*BDCA2-DTR* mice) (Wohn et al, 2013). This study showed that pDC depletion did not affect the severity of skin inflammation. Because of these published results, we had not performed these experiments in our original paper. However, for completion, we now repeated this experiment with *BDCA2-DTR* mice, and treated these mice with imiquimod for 6 days and sacrificed them on d7 (see response to point 3 by Reviewer #2). We did not find any change in the phenotype by pDC depletion as previously published (Wohn et al, 2013). We included these data in Supplementary Fig. 2. K-M and mention them in the main text on p.10 and 11.

5) The statement that "The number of intraepidermal Langerin-negative infiltrating DCs as well as T cells were slightly increased in Lan+ DC-depleted compared to Lan+ DC-sufficient DKO mice" should be omitted since there is no statistical significance. There is no such thing as slightly statistically significant. (And of course, even a statistical significant difference doesn`t equate to biological importance).*

We have now repeated the experiment once more and can show statistical significance for Langerin-negative infiltrating CD11c^{+} APCs (CD45^{+}CD11c^{+}Langerin^{neg}). We have included the new graph in Supplementary Fig. 3L and furthermore describe our finding on p.12 of the results section and on p.18/19 of the discussion. Influx of inflammatory DCs is an important hallmark of psoriasis, since in psoriatic lesions of patients, DCs as TNF-α and iNOS-producing DCs (Tip-DCs) are present in high quantity (Lowes et al, 2005), and are responsible for the production of these two pro-inflammatory mediators.

According to the suggestions of Reviewer #2 (point 4), we performed a more detailed analysis of the T cell compartment in DKO* mice harboring or lacking LCs and found that while γδ T cell numbers were not altered in DKO* mice when compare to control, there was a trend towards increased infiltration of mostly CD4^{+}, but also CD8^{+} TCRβ^{+} T cells. We have added a graph representing these data in Supplementary Fig. 3N, and have described and reformulated our result and conclusion on p. 12 of our manuscript. We have furthermore included the relevant antibodies in Supplementary Table 1.

6) The authors make a fuss about LC depletion resulting in massive IL-23 increase, as emphasized in the abstract). Yet, looking at Fig 7C, the increase in IL-23 comparing LC depleted and non-depleted DKO appears not to be significant!

We have now repeated these experiments with more mice and reanalyzed the original qRT-PCRs data and can demonstrated that IL-23 is significantly increased in LC-depleted DKO* mice (shown in new Fig. 7C). As requested by Reviewer # 2, we have also repeated the IL-23 analyses with more pDC-depleted DKO* mice. While IL-23 levels were again not significantly reduced in the epidermis of pDC-depleted DKO* mice (Fig. 7E), we can now show significant IL-23 reduction in dermal suspensions of DKO* mice upon pDC depletion. Since pDCs are more abundantly present in the dermis, this might reflect the fact that the dermis is also their site of action in regulating IL-23. We have included this set of data in Fig. 7F and describe them on p.16 in our manuscript.

7) Likewise anti-IL-23R treatment of DKO mice in the chronic phase of disease (d14-18) seems not to result in a significant reduction in disease score as compared to untreated DKO mice (Fig 7F). In their interpretation the authors compared a-IL-23R treated DKO at day 28 with untreated and DKO mice at day 14, which is insufficient. How often has this experiment been performed with similar results?

Data in this panel was pooled from two experiments, with a total of n=14 (DKO*mIgG1) and n=9 (DKO* anti-IL-23R) mice. Moreover, the way we originally presented the data was misleading and we would like to apologize for this. We have now rearranged the data presentation and have juxtaposed the various treatments (previously Fig. 7G, now Fig. 7H). We compared the disease

scores of isotype- and anti-IL23R treated DKO* mice on d14 and on d28 and can show that anti-IL23R treatment leads to significant disease amelioration at day 28. The statistical analysis was performed using a Wilcoxon-signed-rank test. At d28 the p value of the phenotype score of anti-IL23R-treated DKO* mice tested before and after treatment was 0.004, presented as **. We now also include statistical analysis of the treatment groups mouse IgG1 versus anti-IL23R treated DKO* mice. This analysis was performed by Mann-Whitney-U test resulting with a P-value of p=0.035, depicted as # in Fig. 7H.

8) The explanation for the differences in skin-resident and bone-marrow derived LCs in chronic (absence of Jun/JunB) vs non-chronic (imiquimoid treated) as determined in the bone marrow chimeras appears to me not really satisfactory. Mice were analyzed at day 9 (DKO) and day 8 (imiquimod-treated), which represents more or less the peak of disease when imiquiomod treatment is continued until then. There are probably differences in molecular requirements for development of psoriasis in the imiquimod vs Jun/JunB-deficiency model rather than simply one being chronic and the other not. I would suggest to extend imiquimod-treatment for another 7 days until day 14, in this case it could be considered as a chronic model and LCs play a role.

Since the experiment proposed by this referee would have considerably delayed the publication of our manuscript without considerably enhancing its scientific impact, the referee, when contacted, proposed the following: „This experiment is not absolutely essential for the main conclusion of the manuscript, but in its absence the authors have to tone down their explanation about the observed differences in LC dynamics and consider the possibility that the molecular pathways for the disease in the two models are not alike."

We are thankful to this reviewer for his/her response and we have accordingly modified the text in the results section on p. 14, that now reads: "In contrast, topical Imiquimod treatment did not result in substantial recruitment of bone-marrow derived LCs (Fig. 6I-L). These results demonstrate that in psoriasis-like disease of DKO* mice, a considerable fraction of LCs are derived from the bone marrow". Additionally, we mentioned the differential observation in DKO* mice versus Imi-treated mice in the discussion on p. 18.

9) Figure 2 shows that DKO mice develop psoriasis peaking at d14. The authors state that this is associated with increased numbers of DCs in the lesions. However, shown are frequencies and not total numbers.

We agree with the referee and have changed it accordingly throughout the text.

10) Gates set for characterization and quantitation for dermal DCs in sup. Fig 1C are questionable. The gated population may consist of (at least) two populations including CD11c-negative (or low) and CD11c-intermedaite cell (both MHC class II positive). Cells should be stained with CD64 and F4/80 to distinguish tissue macrophages, DCs and inflammatory CD11c+ monocyes.

We agree that CD11c and MHC-II coexpression is exhibited by various subtypes of myeloid cells in the dermis, namely CD11b^{+}DCs, moDCs and macrophages (Tamoutounour et al, 2013). According to the referee's suggestions we now performed additional stainings allowing a more detailed analysis of the myeloid compartment in the epidermis and the dermis of d7 and d14 DKO* mice. We quantified CD11b^{+} DCs as well as two different subsets of monocyte-derived DCs and macrophages that differ by expression of MHC-II. This experiment revealed that already at an early timepoint after Tx administration (d7), the frequency of dermal myeloid CD11c^{+} MHC-II^{+} cells as moDCs and MHC-II^{+} macrophages is increased, which remains constant until d14. Interestingly, the frequency of dermal CD11b^{+} DCs is not significantly changed in DKO* mice. In contrast, in the epidermis, myeloid cell-mediated inflammation with a similar composition as in the dermis takes place but is delayed and only present at d14, when inflammatory hallmarks of psoriasis are already morphologically obvious. These data can be found in Fig. 2B, and the description to them was added to the revised manuscript on p. 8. The changes in the Figure legends and text have been underlined.

Furthermore, a sentence referring to early presence of macrophages has been added to the discussion section on p.20. and the experimental procedure of gating and isolation is mentioned in the Methods section on p.24. Relevant antibodies and clones have been added to Supplementary Table 1. The gating strategy is additionally included as Attachment 3 for the reviewer. We are also happy to include them in the manuscript if requested.

11) Frequency of CD11+ cells in the epidermis and dermis go up from d7 to d14. Yet LCs (Langerin+ cells in the epidermis) go down from d7 to d 14. Given that the large majority of CD11c+ cells in the epidermis are LCs, this observation does not make much sense.

While in healthy epidermis LCs constitute the major fraction of DCs, in inflamed epidermis of DKO* mice, many Langerin^{neg} CD11c^{+} MHC-II^{+} cells are present. As already mentioned in the answer to question 5 of Reviewer #2, this is an important hallmark of psoriasis, since in psoriasis patient lesions, DCs as TNF-α and iNOS-producing DCs (Tip-DCs) are present in high quantity (Lowes et al, 2005), and are responsible for the production of these two proinflammatory mediators. Finally, many of the myeloid cell subsets shown in Fig. 2B exhibit CD11c expression (see question 10 by this referee). Additionally, we have included a quantification of the frequency of CD45^{+}CD11c^{+}MHC-II^{+}Langerin^{neg} cells in the epidermis of ears of *jun/junB$^{f/f}$* and d7/14 DKO* mice as determined by flow cytometry which is depicted in Supplementary Fig. 1G and described in the manuscript on p. 9.

12) Fig 2A, C,H, J show percentages, but it is not clear of what. Percentages of live CD45+ showed be shown. Given the low frequency of cells, especially pDCs and LCs, an experiment showing orginal FACS data with gating strategy, i.e. FSC vs SSC -> single cells > live cells -> CD45. And the autofluoresence should be gated out

All flow cytometric gatings were performed by gating live cells following exclusion of doublets. We have now updated the Methods section with information on flow cytometric gating on p.24. Furthermore, the sentence "Flow cytometric quantification is depicted as percentage of live cells" has been added to the legend of Fig. 2,4 5, 6, 7 and Supplementary Fig. 2, 3, and 5. Additionally, we have included an example of our flow cytometric gating, as requested by this referee (Attachment 4) to be sent to the referees and the editor. We are also happy to include them in the manuscript if requested.

2nd Editorial Decision 04 August 2014

Thank you for the submission of your revised manuscript to EMBO Molecular Medicine. We have now received the enclosed reports from the two Reviewers who were asked to re-assess it. As you will see they are now globally supportive and I am pleased to inform you that we will be able to accept your manuscript pending editorial final amendments.

Please submit your revised manuscript within two weeks. I look forward to seeing a revised form of your manuscript as soon as possible.

***** Reviewer's comments *****

Referee #2 (Remarks):

This reviewer is satisfied with the revisions done by the authors.

Referee #3 (Comments on Novelty/Model System):

Compared to the initial review, I have upgraded novelty of the results from medium to high, because the authors now provide data suggesting an IL-17/IL22 independent effector mechanism mediated by LC in this model of psoriasis.

Referee #3 (Remarks):

The manuscript has been substantially improved by providing stronger data on the role of IL-23 independent of IL-17A,F and IL-22 in this psoriasis model.
The axis of figures 6c,e,f must be correctly labelled with il23p19(or il23a) mRNA instead of IL-23 mRNA.
